# Supplementary material for: Perinatal healthcare for women at risk of children’s social care involvement: a qualitative survey of professionals in England
Source: BMJ Open. 2024 Mar 5;14(3):e082914. doi: 10.1136/bmjopen-2023-082914 (PMC10916114; doi:10.1136/bmjopen-2023-082914)
Supplement: Supplementary data [file bmjopen-2023-082914supp001.pdf]

**Supplementary Material 1**

# Supporting women with complex health needs who might be at risk of children's social care involvement – survey questions

**About you**

The following questions will give us a better understanding of who you are and your role in maternity services. Please skip any questions you don't feel comfortable answering.

**Gender identity**

- Male
- Female
- Non-binary
- Other
- Prefer not to say

**Age**

- 18-14
- 25-29
- 30-24
- 35-39
- 40-44
- 45-49
- 50-54
- 55+
- Prefer not to say

**Ethnicity**

- Asian/Asian British (Indian, Pakistani, Bangladeshi, Chinese, other Asian background)
- Black, Black British, Caribbean or African (Caribbean, African, Any other Black, Black British, or Caribbean background)
- Mixed or multiple ethnic groups (White and Black Caribbean, White and Black African, White and Asian, Any other Mixed or multiple ethnic background).
- White (English, Welsh, Scottish, Northern Irish or British, Irish, Gypsy or Irish Traveller, Roma, Any other White background)
- Other ethnic group (Arab, Any other ethnic group)
- Prefer not to say

**What is your job role within maternity services?**

- Midwife
- Health visitor
- Support worker

- Staff nurse
- Neonatal nurse
- Obstetrician
- Perinatal psychologist
- Perinatal psychiatrist
- Other (please specify)

**What setting do you work in? (select as many as apply)**

- NHS/public sector
- Private sector healthcare
- Third sector/charity organisations

**In which region of England do you currently work?**

- North West
- North East
- Yorkshire and the Humber
- East Midlands
- West Midlands
- South East
- East of England
- South West
- Greater London

**How long have you been in this field?**

- Less than 1 year
- 1-2 years
- 2-5 years
- 5-10 years
- 10-15 years
- 15 years+

**Briefly describe your job role**

E.g. 'I work as a health visitor and support families with a new baby from pregnancy until the child is 5 years old. I've been in the same clinic in Manchester for 10 years and specialise in supporting women breastfeed. I have recently started facilitating a breastfeeding 'drop-in' clinic once a week for mothers.'

**Your experiences and attitudes**

***Chronic physical conditions***

We define chronic physical conditions as conditions that last 1 year or more and require ongoing medical attention or limit activities of daily living or both (e.g., epilepsy, asthma, heart-disease).

Have you had experience supporting women with chronic physical conditions in maternity services?

- Yes
- No
- Unsure

If yes, what did you do in your role to support these needs?

What are some of the barriers to providing adequate maternity care to women with chronic physical health conditions?

Tell us how much you agree with the following statements.

I have had appropriate training to know how to support women with chronic physical conditions.

- Strongly disagree
- Disagree
- Neither agree nor disagree
- Agree
- Strongly agree

I feel confident supporting women with chronic physical conditions in maternity services.

- Strongly disagree
- Disagree
- Neither agree nor disagree

- Agree
- Strongly agree

***Mental health need***
We define mental health need as the presentation, or diagnosis, of symptoms associated with depression, anxiety, panic, mania, delusions, hallucinations or disordered behaviours (i.e., eating, checking, compulsions).

Have you had experience supporting women with mental health needs in maternity services?

- Yes
- No
- Unsure

If yes, what did you do in your role to support their needs?

What are some of the barriers to providing adequate maternity care to women mental health needs?

Tell us how much you agree with the following statements.
I have had appropriate training to know how to support women with mental health needs in maternity services.

- Strongly disagree
- Disagree
- Neither agree nor disagree
- Agree
- Strongly agree

I feel confident supporting women with mental health needs in maternity services.

- Strongly disagree
- Disagree
- Neither agree nor disagree
- Agree
- Strongly agree

***Developmental disabilities***

We define developmental disabilities as an impairment in one of four areas: physical, learning, language or behaviour. Developmental disabilities are inclusive of intellectual (or learning) disabilities, attention deficit and hyperactivity disorder (ADHD) and autism spectrum disorders (ASD).

Have you had experience supporting women with developmental disabilities in maternity services?

- Yes
- No
- Unsure

If yes, what did you do in your role to support these needs?

| |
|---|
| |

What are some of the barriers to providing adequate maternity care to women with developmental disabilities?

| |
|---|
| |

Tell us how much you agree with the following statements.
I have had appropriate training to know how to support women with developmental disabilities in maternity services.

- Strongly disagree
- Disagree
- Neither agree nor disagree
- Agree
- Strongly agree

I feel confident supporting women with developmental disabilities in maternity services.

- Strongly disagree
- Disagree
- Neither agree nor disagree
- Agree
- Strongly agree

***Substance misuse***

We define substance misuse as the use of alcohol, illegal drugs, or over-the-counter or prescription medications in a way that is harmful to the user or those around them. Have you had experience supporting women with substance misuse challenges in maternity services?

- Yes
- No
- Unsure

If yes, what did you do in your role to support these needs?

What are some of the barriers to providing adequate maternity care to women with substance misuse challenges?

Tell us how much you agree with the following statements.

I have had appropriate training to know how to support women with substance misuse challenges.

- Strongly disagree
- Disagree
- Neither agree nor disagree
- Agree
- Strongly agree

I feel confident supporting women with substance misuse challenges.

- Strongly disagree
- Disagree
- Neither agree nor disagree
- Agree
- Strongly agree

***Child protective services***

Supporting women at risk of child protection involvement. Child protective services may become involved with a family when there are concerns around parenting capacity, or it is believed that the child is suffering neglect or abuse. The antenatal

period is a crucial time for intervention and support for birth mothers at risk of child protection involvement, including those with complex health needs.

Have you had experience supporting women with child protection involvement in maternity services?

- Yes
- No
- Unsure

If yes, what did you do in your role to support these needs?

What are some of the barriers to providing adequate maternity care to women with child protection involvement?

Tell us how much you agree with the following statements.

I have had appropriate training to know how to support women with child protection involvement.

- Strongly disagree
- Disagree
- Neither agree nor disagree
- Agree
- Strongly agree

I feel confident supporting women with substance misuse challenges.

- Strongly disagree
- Disagree
- Neither agree nor disagree
- Agree
- Strongly agree

***Example situation***

I would like you to think of a time where you felt you might need to make a referral to children's social care [or to your internal child safeguarding team] as a women's pregnancy progressed.

Describe what made you worried about the child and/or family. Please refrain from using patients' names or identifiable features.

Are there any other comments you would like to make about supporting women with complex health needs in maternity services?

End of the survey.
