# Supplementary material for: Perinatal healthcare for women at risk of children’s social care involvement: a qualitative survey of professionals in England
Source: BMJ Open. 2024 Mar 5;14(3):e082914. doi: 10.1136/bmjopen-2023-082914 (PMC10916114; doi:10.1136/bmjopen-2023-082914)
Supplement: Author's manuscript [file bmjopen-2023-082914.draft_revisions.pdf]

BMJ Open is committed to open peer review. As part of this commitment we make the peer review history of every article we publish publicly available.

When an article is published we post the peer reviewers' comments and the authors' responses online. We also post the versions of the paper that were used during peer review. These are the versions that the peer review comments apply to.

The versions of the paper that follow are the versions that were submitted during the peer review process. They are not the versions of record or the final published versions. They should not be cited or distributed as the published version of this manuscript.

BMJ Open is an open access journal and the full, final, typeset and author-corrected version of record of the manuscript is available on our site with no access controls, subscription charges or pay-per-view fees (http://bmjopen.bmj.com).

If you have any questions on BMJ Open's open peer review process please email info.bmjopen@bmj.com

BMJ Open

Perinatal healthcare for women at risk of children's social care involvement: a qualitative survey of professionals in England

Journal:	*BMJ Open*
Manuscript ID	bmjopen-2023-082914
Article Type:	Original research
Date Submitted by the Author:	06-Dec-2023
Complete List of Authors:	Grant, Claire; University College London Institute of Epidemiology and Health Care, Department of Epidemiology and Public Health<br>Bicknell-Morel, Tamsin; Homerton Healthcare NHS Foundation Trust<br>Lever Taylor, Billie ; King's College London Florence Nightingale Faculty of Nursing Midwifery & Palliative Care, Division of Methodologies<br>Powell, Claire; University College London, Institute of Child Health<br>Blackburn, Ruth; University College London Great Ormond Street Institute of Child Health, Population, Policy and Practice<br>Lacey, Rebecca; St George's University of London, Population Health Research Institute; University College London, Department of Epidemiology and Public Health<br>Woodman, Jenny; University College London Social Research Institute, Thomas Coram Research Institute
Keywords:	Child protection < PAEDIATRICS, MENTAL HEALTH, Maternal medicine < OBSTETRICS, Substance misuse < PSYCHIATRY

SCHOLARONE™
Manuscripts

I, the Submitting Author has the right to grant and does grant on behalf of all authors of the Work (as defined in the below author licence), an exclusive licence and/or a non-exclusive licence for contributions from authors who are: i) UK Crown employees; ii) where BMJ has agreed a CC-BY licence shall apply, and/or iii) in accordance with the terms applicable for US Federal Government officers or employees acting as part of their official duties; on a worldwide, perpetual, irrevocable, royalty-free basis to BMJ Publishing Group Ltd ("BMJ") its licensees and where the relevant Journal is co-owned by BMJ to the co-owners of the Journal, to publish the Work in this journal and any other BMJ products and to exploit all rights, as set out in our licence.

The Submitting Author accepts and understands that any supply made under these terms is made by BMJ to the Submitting Author unless you are acting as an employee on behalf of your employer or a postgraduate student of an affiliated institution which is paying any applicable article publishing charge ("APC") for Open Access articles. Where the Submitting Author wishes to make the Work available on an Open Access basis (and intends to pay the relevant APC), the terms of reuse of such Open Access shall be governed by a Creative Commons licence – details of these licences and which Creative Commons licence will apply to this Work are set out in our licence referred to above.

Other than as permitted in any relevant BMJ Author's Self Archiving Policies, I confirm this Work has not been accepted for publication elsewhere, is not being considered for publication elsewhere and does not duplicate material already published. I confirm all authors consent to publication of this Work and authorise the granting of this licence.

For peer review only

Perinatal healthcare for women at risk of children's social care involvement: a qualitative survey of professionals in England

Author list: Claire Grant[1], Tamsin Bicknell-Morel[2], Billie Lever Taylor[3], Claire Powell[4], Ruth Blackburn[4], Rebecca Lacey[1,5], Jenny Woodman[6]

Department of Epidemiology and Public Health, University College London, UK[1]
Homerton Healthcare NHS Foundation Trust, London, UK[2]
Division of Methodologies, Florence Nightingale Faculty of Nursing, Midwifery & Palliative Care, Kings College London[3]
Population, Policy and Practice Department, UCL Great Ormond Street Institute of Child Health, London, UK[4]
Population Health Research Institute, St George's, University of London, London UK[5]
Thomas Coram Research Unit, Social Research Institute, Institute of Education, University College London, UK[6]

Corresponding author

Ms Claire Grant,
Department of Epidemiology and Public Health
University College London
1-19 Torrington Place, London WC1E 6BT
claire.grant.20@ucl.ac.uk

Word count

3,976

Key words

Perinatal health, child protection, maternity

For peer review only

Abstract

Background

Women with complex health needs are more at risk of having children's social care involvement with their newborns than other mothers. Around the time of pregnancy, there are opportunities for health services to support women with these needs and mitigate the risk of mother-baby separation. Yet little is known about healthcare professionals' experiences of providing this support.

Methods

We administered an online survey to perinatal healthcare professionals across England (n=70 responders), including midwives, obstetricians, perinatal psychologists/psychiatrists, and health visitors. We asked about their experiences of providing care for pregnant women with chronic physical conditions, mental health needs, intellectual/developmental disabilities and substance use disorders, who might be at risk of children's social care involvement. We conducted a framework analysis.

Results

We constructed five themes from participant data. These include, 1) inaccessible healthcare for women with complex needs, 2) the challenges and importance of restoring trust, 3) services focusing on individuals, not families, 4) the necessity and caution around multidisciplinary support, and 5) underfunded services inhibiting good practice.

For peer review only

Conclusions

Women who are at risk of children's social care involvement will likely experience perinatal healthcare inequities. Our findings suggest that current perinatal healthcare provision for this population is inadequate and national guidelines need updated to inform support.

For peer review only

Article summary

What is already known on this topic?

Women with complex health needs have an increased risk of children's social care involvement in the perinatal period. During this time, healthcare professionals have opportunities to support women and mitigate the risk of mother-baby separation.

What this study adds?

Our findings provide insight into experiences of supporting this population among perinatal healthcare practitioners in England. Respondents described fostering trust with mothers, responding to whole-family needs, and working in a holistic and multidisciplinary way, as key to providing adequate, equitable care. Yet overwhelmingly, healthcare professionals felt unable to deliver this care in services and systems which were not equipped.

How this study might affect research, practice or policy?

Our findings can be used to inform service and policy level changes for supporting women at risk of children's social care involvement. National guidelines should be updated to improve perinatal healthcare access and outcomes for this population.

Strengths and limitations of this study

- This study offers a timely insight into lived experiences of providing perinatal healthcare for women at risk of children's social care involvement.
- We collected survey data across different local authorities in England and across a range of professionals who support pregnant women in healthcare.

For peer review only

- Free-text responses allowed professionals to share in-depth responses and case examples of service provision and service constraints.
- Findings can contribute towards the development of national guidelines for supporting women at risk of children's social care involvement.

For peer review only

Background

From conception until one year after birth (the 'perinatal' period), mothers in England have various routine contacts with healthcare services. As well as labour and birth support, most women are offered up to 10 antenatal appointments to check the health and development of their babies, in addition to regular postnatal reviews from health visitors, and specialist input, such as from perinatal mental health services, as needed. (1)

Mothers with complex health needs are more likely to have complications during pregnancy and childbirth. (2–4) Newborns are directly affected by parental health and health behaviours, via in utero effects to the developing baby and post-natal caregiving practices. (5) There are also indirect harms of poor parental health on infants, including unstable housing, compromised parental capacity, and increased risk of child abuse/neglect. (6) We know that histories of trauma, experiences of chronic stress, and psychosocial adversities can impact on women's experiences of birth and infant outcomes. (7,8) Women who experience severe and multiple disadvantages can be underserved by maternity services and overrepresented in children's social care. (9,10)

In a 2022 review of perinatal mortality in the UK, 20% of women who died during pregnancy, or in the year following, were found to be involved with children's social care. (11) Maternal serious mental illness (12), substance use disorder, (13) and intellectual/developmental disability (14), have all been associated with an increased risk of pre-birth assessments and newborn entry into out-of-home care. (10) The fear of children's social care involvement can itself act as a barrier for parents accessing help from relevant services, including specialist support for their mental health or substance

For peer review only

use. (15,16) Additionally, health needs can co-exist and be compounded by adverse social circumstances, such as low social support and living in poverty. (17,18)

Many of the challenges faced by women with children's social care involvement are the product of lifelong disadvantages and lack of meaningful support (19), factors which long pre-date their pregnancy. (7) There are also various barriers to providing adequate care for this population, including parental distrust of services. (15) Nevertheless, professionals working in perinatal healthcare have opportunities to support women in services, to mitigate some of the health risks associated with children's social care involvement, and to make appropriate referrals for continued support. (20) Our study aims to explore the experiences of perinatal healthcare providers and reflect on how services and systems might better support women at risk of children's social care involvement (i.e., those with 'complex needs').

Methods

Survey methods were chosen due to their ability to efficiently collect qualitative data across settings and populations. The anonymity of completing the survey online was also thought to increase honest accounts of professionals' experiences, attitudes, and beliefs. (21)

Sampling strategy

Healthcare professionals were invited to participate via email, newsletters, and social media posts on Twitter between October-December 2022. The recruitment process utilised existing clinical and academic networks, including maternity interest groups and relevant organisations. Participants had to be employed in a healthcare role within

For peer review only - http://bmjopen.bmj.com/site/about/guidelines.xhtml

perinatal services in England. All responses were voluntary, and consent was sought from each participant. Ethical approval was obtained from UCL Research Ethics Committee (ref: 21605/001) and data were collected via Qualtrics. (22)

Survey questions

We developed our survey questions based on findings from a systematic scoping review into parental health in the context of children's social care. (10) We asked about experiences of supporting women with different types of complex health needs that have been associated with children's social care involvement. These included chronic physical conditions, mental health needs, intellectual/developmental disabilities, and substance use disorders (see Table 1 for definitions). Participants were asked to reflect on their experiences of supporting women with these needs. Most survey questions were free text and had no limit on the length of participants' responses. We asked respondents what they did in their role to support these women and what the barriers to providing care were. We also asked about experiences of safeguarding and referrals to children's social care (see Supplementary Material 1 for survey questions).

Table 1 Definitions of health need

**Mental health need**	The presentation or diagnosis of symptoms associated with depression, anxiety, psychosis, eating disorders, and emotional distress (including post-traumatic stress disorder).
**Developmental disabilities**	An impairment of learning, language, or behaviour, inclusive of intellectual (or learning) disabilities, attention deficit and hyperactivity disorder (ADHD) and autism spectrum disorders (ASD).
**Substance use disorders**	The use of alcohol, illicit drugs or over the counter or prescription medications in a way that is harmful to the user or those around them.

For peer review only - http://bmjopen.bmj.com/site/about/guidelines.xhtml

**Chronic physical conditions**	Conditions that lasted 1 year or more and required ongoing medical attention or limited activities of daily living or both (e.g., epilepsy, permanent injuries).

Analysis

Descriptive statistics of demographic data were calculated using StatsiQ within the Qualtrics software. Free-text responses were treated as qualitative data and analysed using framework analysis. (23) Authors familiarised themselves with the free-text responses and conducted an initial inductive coding exercise with a subset of data. Phrases were labelled to capture meaning or content relevant to the research question. Initial codes were reviewed and used to develop an analytical framework. Once the framework was refined, all free-text responses from the survey were exported into Excel and data organised within the framework to relevant codes. Codes were conceptually organised to construct themes and capture participant experiences.

Patient and public involvement

Researchers presented findings to two women with lived experience of children's social care involvement. We asked women to reflect on our themes and descriptions in relation to their own experiences. Minutes from this meeting was considered part of data interpretation and their commentary is included in the results.

Participants

A total of 70 professionals responded to our online survey, with most participants identifying as female (96%) and White (88%). Half of the respondents worked as midwives (50%) and around half worked in Greater London (46%). See Table 2 for distribution of responders across England. Nearly all had experience of supporting

For peer review only

women with mental health conditions (96%), and around three quarters had supported mothers with substance use disorders (78%), and intellectual/developmental disabilities (72%). Over half had supported a woman with a chronic physical condition (53%) and nearly all had supported a pregnancy that had children's social care involvement (92%) (See Table 2).

Table 2 Participant demographics

		Count (n)	Percentage (%)
Healthcare professionals			
**Gender**			
	Female	67	96
	Male	3	4
**Age**			
	18-29	19	27
	30-39	18	26
	40-49	17	24
	50+	16	23
**Ethnicity**			
	Asian/Asian British	3	5
	Black, Black British, Caribbean, or African	4	5
	White	61	88
	Prefer not to say	2	2
**Job Role**			
	Midwife	35	50
	Health visitor	8	12
	Nurse or support worker	4	6
	Perinatal psychologist/psychiatrist	7	10
	Other	16	22
**Region of England**			
	North East	1	2
	Yorkshire and the Humber	10	15
	East Midlands	4	6
	West Midlands	11	16
	South East	6	9
	East of England	2	3
	South West	2	3
	Greater London	31	46

For peer review only - http://bmjopen.bmj.com/site/about/guidelines.xhtml

**Years of experience**			
	Less than 1	11	16
	1-5 years	14	20
	5-10 years	20	29
	10-15 years	10	15
	15 years +	10	15
	Prefer not to say	3	5
**Direct experience supporting**			
	Chronic physical conditions	37	53
	Mental health difficulties	67	96
	Substance use disorders	54	78
	Developmental disabilities	50	72
	Child safeguarding concerns	64	92

Results

From participant data, we constructed five themes, 1) inaccessible healthcare for women with complex needs, 2) the challenges and importance of restoring trust, 3) services focusing on individuals, not families, 4) the necessity and caution around multidisciplinary support, and 5) underfunded services inhibiting good practice. Descriptions of each theme and supporting quotations are reported below, alongside commentary from our lived experience group.

1. Inaccessible healthcare for women with complex needs

Participants described challenges providing equitable perinatal healthcare for women with complex needs. They gave examples of women with mental health difficulties, disabilities, and chronic conditions receiving care in universal clinics which were not suitable or equipped for their needs.

> "High thresholds for specialist interventions means that the responsibility of ensuring safety of mums and babies is down to us." (ID22, family nurse)

For peer review only - http://bmjopen.bmj.com/site/about/guidelines.xhtml

Professionals adjusted care provision as best they could but felt that this was limited without specialist knowledge or facilities. Respondents felt that mothers who faced multiple adversities, including migrant women, often had more complicated pregnancies yet faced additional barriers to care (e.g., cultural, language, and financial).

"I cared for a woman with a learning disability who was an undocumented migrant…and there was lots of social services involvement around protecting the baby, but none available to support her to keep her child, because of her status." (ID42, midwife)

Midwives stressed the importance of having interpreters at appointments and using 'hospital passports' to aid communication with mothers who had language or communication impairments. These documents contained personalised information for medical staff, including women's care preferences and requirements.

"It's important to use and adapt resources in services, such as birth plans that use images rather than words, or hospital passports to aid communication for those who need it." (ID47, midwife)

Access to specialist services for women was inhibited by lack of local resource, high thresholds, and narrow windows of eligibility. For professionals who had experience practicing in different areas, the disparity in care quality across England was noted. Participants also described a rise in the number of women using substances throughout pregnancy. They felt that available support was insufficient, especially given that substance use was a prominent feature of safeguarding and risk reports.

For peer review only - http://bmjopen.bmj.com/site/about/guidelines.xhtml

"There is a real stigma in both the public and also in maternity services for pregnant women who use substances. Some services don't even have a specialist substance misuse midwife." (ID07, midwife)

Respondents reflected on how women who used substances were ineligible for support at a mental health 'mother and baby unit' (MBU), despite mental health needs and substance use likely co-existing. Women were also excluded from perinatal mental health services if they were under 18, had experienced a pregnancy loss (stillbirth, miscarriage, or termination) or had their baby removed at birth.

"My clients are mainly under 18, which means that they are unable to access perinatal mental health services and get placed into CAMHS (child and adolescent mental health services) which is generally inappropriate for their needs." (ID39, Family Nurse)

Other services, such as health visiting, halted following newborn removal via children's social care. Professionals commented on this care as fragmented and shortsighted. Our lived experienced group emphasised the importance of being able to access perinatal care, even when those babies were no longer living or had been placed in out of home care.

"The timeframe they have is so short and once discharged have very little parallel planning for future treatment and support." (ID44, midwife)

Making service provision accessible often required flexibility, such as options for longer or last-minute appointments, communication via SMS, or the option of home-based support. There was consensus across professional groups that flexible, and women

For peer review only - http://bmjopen.bmj.com/site/about/guidelines.xhtml

centered perinatal care was best practice, yet professionals reported feasibility challenges implementing such strategies within NHS settings.

2. The challenges and importance of restoring trust

Respondents stated that building strong relationships with women was key to delivering meaningful and equitable care. This included providing emotional support, being an advocate, and treating women with dignity and respect. Our lived experience group felt that professionals should show kindness in their care and be compassionate towards women's circumstances. For psychologists and perinatal mental health therapists, fostering trusted clinical relationships was considered a key feature of treatment and service delivery. Yet the ability to foster trust with families was not always possible for professionals. Midwives described busy caseloads and staffing issues as barriers to establishing trust with mothers who had complex needs, particularly when there was lack of continuity of care. All professionals acknowledged the impact of a positive relationship on engagement with services and mother/baby outcomes.

"Relationship building is the key. A woman will return for advice if she feels understood and listened to. I am in a much better position to support and advise if a woman trusts me and understands what I say to her." (ID11, health visitor)

For women at risk of children's social care involvement, professionals felt there was an ingrained distrust of services. Respondents reflected on having to restore women's confidence in public services' ability to support them. Considerations of how to build back trust included validating women's experiences, providing a non-judgmental space

For peer review only - http://bmjopen.bmj.com/site/about/guidelines.xhtml

to listen, and understanding aspects of appointments which might be difficult (e.g., discussing safeguarding concerns and potential outcomes).

“Remaining open and non-judgemental to women. To remain a person that they can trust and to help anchor them into health care services. Support them with social care processes whilst giving them realistic expectations of what is likely to happen.” (ID62, midwife)

Our lived experience group agreed that honest and clear communication helped with any escalation of safeguarding concerns.

Participants stated the importance of being able to provide support for families and not just appearing to be policing them. They stated that if a family do not feel they are being heard or supported, they will be more likely to withhold information about health behaviours that trigger concern, such as substance use. Yet the support professionals provided was constrained by service rigidity and safeguarding policies. Some midwives provided examples of strained services being risk averse and inhibiting meaningful risk management with families experiencing difficulties. Referrals to internal safeguarding teams, domestic abuse pathways, or children’s social care, at times lead to further distrust of public services for families already under stress.

“A woman disclosed domestic violence and although I explained that I had to make a referral I was worried that I would lose her trust. This is what happened - she was contacted by social services in a very unhelpful way and was furious. She declined any further care from me.” (ID33, midwife)

For peer review only

Responding to safeguarding concerns sensitively and proportionately relied on trusted clinical relationships between professionals, as well as with families.

"Often, we are not getting feedback on safeguarding referrals, the information all seems to flow one way. So, we are not always feeling that our voices, as midwives, are being heard when social services get involved." (ID39, midwife)

3. Services focusing on individuals, not families

Supporting the health of an unborn baby was a main consideration for determining maternal care plans and pathways, often limiting more holistic, family-focused work.

"Services may or may not take a rounded view of the mother and her child's need." (ID22, psychologist), as "the unborn baby or child's safety can often take priority over efforts to safeguard the family as a whole." (ID54, midwife)

A participant (psychologist) stated that services should strive to support both mother and baby, offering services that target women's needs as a means of enabling caregiving capacity, and therefore supporting newborn safety. Midwives and health visitors commented on the importance of looking to existing networks of support in a woman's life, e.g., partner, family, or friends, to support risk management. Involving partners was described as integral to crisis planning, yet support became more complicated when needs were interrelated (e.g., instances of domestic abuse or co-habiting drug use). Participants described how household risks were often not within perinatal service remits, despite raising safeguarding concerns for the baby.

For peer review only - http://bmjopen.bmj.com/site/about/guidelines.xhtml

"Often safeguarding concerns are related to fathers' needs. A woman's partner having severe mental health problems, not engaging with services, being abusive in the house and the woman being subject to coercive control." (ID62, clinical nurse specialist)

Our lived experience group reflected on how services should be mindful of any potential abuse in households and work to support the whole family in a way that doesn't further the risk to the mother. One of the health visitors commented on the dearth of services available for birth fathers with complex health needs. Whilst, in principle, some perinatal services supported both parents, all were targeted at mothers and most had little capacity to support the wider family unit.

For some women, their own childhood experiences and relationships with their parents played a negative role in their transition to motherhood. Two of the psychology respondents described the perinatal period as a sensitive time for managing complex trauma and felt services should be equipped to manage this.

"There are not enough resources to help women with severe trauma through the several years needed to make it safe/ viable for them to be good and creative mothers to their children when they had insufficient support themselves when a child." (ID22, psychologist)

These participants recommended that any 'whole family' support considers both families of origin and families of creation. Our lived experience group also commented on services acknowledging children in families who are no longer alive or have been placed in out-of-home care.

4. The necessity and caution around multidisciplinary support

For peer review only - http://bmjopen.bmj.com/site/about/guidelines.xhtml

Participants reported liaising and working alongside other professionals to support women with complex needs yet described challenges to shared care plans.

"Social services use a different system to us, so information sharing is not always done timely. It's even difficult to find schedules for all to be available at the same time." (ID03, occupational therapist)

However, participants cautioned that routine data sharing across sectors inhibited mothers' engagement with services, due to concern that information was being communicated to children's social care.

"Pressure from other services, such as children's social care, make it difficult for women to engage meaningfully with mental health services, making the support superficial. There is fear that services feedback to social care and influence child protection proceedings." (ID52, staff nurse)

Participants identified high thresholds for specialist services as a barrier to multidisciplinary working, with referrals being rejected or mothers being added to long waiting lists. Midwives referenced specialist mental health services as especially hard to access, with many women left without support throughout their pregnancy.

"I have had a lot of women struggle to access mental health services due to long waiting lists, sometimes longer than their pregnancies. I have also had many women rejected because their mental health condition was not severe enough." (ID48, midwife)

Successfully liaising with housing, benefits, and similar services was viewed as integral to supporting women's health. Respondents mentioned that local and third sector offerings should be a part of all signposting and referral processes. This was

For peer review only - http://bmjopen.bmj.com/site/about/guidelines.xhtml

corroborated by the lived experience group, who appreciated professionals having local knowledge of organisations that might help specific needs. Yet survey responses illustrated how staff often felt unprepared when supporting women in situations, or with conditions, that they had not received training on. Participants acknowledged that services being overstretched meant less time to dedicate to 'over and above' care, such as building a knowledge base of local provision, learning about more unusual conditions, or engaging with optional training. This left staff feeling less able to fulfil their role, for example, some health visitors and midwives described being unable to provide breastfeeding advise to women on certain medications.

"Not everyone understands the full extent of conditions. Staff at services dealing with chronic physical health conditions won't know the impact it has on pregnancy, and maternity staff don't know the full extent of how pregnancy can impact on chronic physical health conditions." (ID36, occupational therapist)

Our lived experience group spoke about the importance of understanding the 'whole picture' and not to simply focus on needs which fit within the remit of a specific service.

5. Underfunded services inhibiting good practice

Most notably, midwives reported insufficient staff levels and an overwhelming number of referrals and appointments. All professionals reported concern around the lack of resourcing for staff and services, alongside an increasing demand of complex pregnancies.

For peer review only - http://bmjopen.bmj.com/site/about/guidelines.xhtml

> "Midwives are all incredibly busy due the national shortage of midwives. Women working with children's social care require additional midwifery input which many feel they do not have capacity to facilitate." (ID22, midwife)

Midwives also felt that training on specific health needs was insufficient, especially for temporary bank staff brought in due to staffing shortages. One participant reflected on training courses focusing on how to refer to specialist midwives, but not how to support these women in regular antenatal clinics. They reported feeling worried about not knowing how to answer mothers' questions or how to best tailor care.

> "Having too many babies and women on our caseloads with specialist needs, without being trained to be able to support them. This is a growing area of occurrence and NHS services need to increase their resources and staffing to meet these growing demands." (ID66, midwife)

Staff across professions and services reported high rates of burn out, with unsustainable staffing levels and expectations. Many reflected on how being unable to adequately support mothers had left them feeling frustrated and disheartened. For some respondents, there were comments on how vicarious trauma had impacted on their own wellbeing and ability to fulfil their duties of care.

> "It is a very emotionally draining job and often can be very sad. There is a lack of adequate resourcing and capacity issues within the services themselves." (ID08, midwife)

Most participants referenced good leadership, management, and clinical supervision as key components to fostering a healthy workforce.

For peer review only

"Effective supervision is one of the key ways in which we can help to keep babies safe and support their mothers." (ID52, psychologist)

Responses spoke to wider fatigue experienced by public service staff navigating public spending cuts and austerity measures.

For peer review only

For peer review only - http://bmjopen.bmj.com/site/about/guidelines.xhtml

Discussion

Our findings describe experiences of perinatal healthcare professionals who support women with complex needs at risk of children's social care involvement. In keeping with wider literature, our results illustrate shortcomings in care provision for women experiencing multiple disadvantages. (9) Given the recent independent review into children's social care, (24) and the push for targeted, early help for vulnerable families, this study offers a timely insight into the role of perinatal services.

Healthcare professionals shared ideas for adaptations and service-level changes that would improve perinatal equity for these mothers, including resourcing professionals to deliver family focused, multidisciplinary care which is compassionate and proportionate to need. This is consistent with what mothers involved with children's social care have reported elsewhere; a need for comprehensive support which addresses multiple disadvantages and enables safe parenting. (20,25,26) Yet despite agreement between mothers and professionals of what 'good' perinatal healthcare looks like, our findings highlight various barriers to implementing this in practice.

Our results indicate that despite the will of individual practitioners, systemic issues within the NHS can result in women at risk of children's social care receiving inadequate perinatal healthcare. (19) Maternity services in England continue to be an area of concern for the Care Quality Commission (CQC), with services and staff under huge pressure to maintain safe clinical standards. (27) In order to provide meaningful support to vulnerable families in the antenatal period, professionals and services must be properly resourced. (28) For example, developing trust with mothers was important for delivering effective care in this context, (29) however professionals described

For peer review only - http://bmjopen.bmj.com/site/about/guidelines.xhtml

challenges doing so in services that were overstretched, understaffed, and managing increasingly complex caseloads. (24) Our results draw attention to the dedication of perinatal healthcare staff who were often doing the best they could, with the resources they had, to meet the needs of vulnerable families. Yet we know that increasing numbers of front-line staff are leaving due to exhaustion and burnout. (30) In keeping with the NHS Long Term Plan, our findings highlight the need to drive recruitment into professional training pathways, such as midwifery and psychology, (31) as well as retain staff in public healthcare, through adequate resourcing, good leadership, and opportunities for professional development. (32)

We found that rigid service structures can leave women who experience multiple disadvantages in the perinatal period falling between the gaps of provision. (9,19) Without targeted intervention during pregnancy, women who are vulnerable to children's social care involvement are not given a fair opportunity to promptly address their difficulties. (9) As illustrated in our findings, this can be frustrating for professionals who often feel unable to provide effective care, or hold family-level risk, within perinatal services. As a result, women can experience fragmented care which is not always responsive to level of need. For example, women being 'left behind' by perinatal services following mother-baby separation, despite the known consequences on maternal health. (10,33,34)

There are local examples of good practice, including continuity of care models, (35) pre-birth support led by specialist midwives, (36) and interventions to support women in perinatal services following newborn separation. (37) However, such initiatives are not underpinned by national policy or guidelines, leading to inconsistent provision for

For peer review only

women across the country. (38) Previously published antenatal guidelines from 2010 (last reviewed in 2018) offer advice for supporting women with ‘complex social factors’. (39) Yet these guidelines do not address multiple and intersectional disadvantages, or the impact of maternal trauma relevant to service provision (9). More recent evidence has indicated that women at risk of children’s social care involvement require targeted and specialist perinatal care (20,40). Our results support the need for updated national guidelines. Some recommendations are outlined in Table 3.

Table 3 Suggested updates to NICE guidelines by research theme

**Research theme**	**Current NICE guidelines**	**Suggested update**
1. Inaccessible healthcare for women with complex needs	“At the booking appointment, give the woman a telephone number to enable her to contact a healthcare professional outside of normal working hours, for example the telephone number of the hospital triage contact, the labour ward or the birth centre.”	a. Service leads to provide oversight on tailored care provision for women experiencing multiple disadvantages, including flexible delivery and continuity of care<br><br>b. To deliver specialist perinatal healthcare in circumstances where mothers ‘fall between the gaps’ of services (e.g., migrant women, mothers <18 years old, those who use substances, or have lost an infant to death or to care).
2. The challenges and importance of restoring trust	“Respect the woman's right to confidentiality and sensitively discuss her fears in a non-judgemental manner.”<br><br>“Addressing women's fears about the involvement of children's services and potential removal of their child, by providing information tailored to their needs”	a. To understand the impact of past and ongoing trauma on families’ interactions with services and remain empathetic when addressing non-engagement<br><br>b. To be transparent about service capabilities and local safeguarding protocols; cultivating systems where vulnerable families feel heard and supported, rather than policed and judged.

For peer review only

For peer review only - http://bmjopen.bmj.com/site/about/guidelines.xhtml

3. Services focusing on individuals, not families	"In order to facilitate discussion of sensitive issues, provide each woman with a one-to-one consultation, without her partner, a family member or a legal guardian present, on at least one occasion."	a. Consider how services and third sector organisations might support 'whole-family' need, including domestic abuse, and birth fathers' mental health or substance use<br><br>b. To work with mothers, and families, to build risk management plans which draw on their strengths and existing networks of support.
4. The necessity and caution around multidisciplinary support	"Consider initiating a multi-agency needs assessment, including safeguarding issues, so that the woman has a coordinated care plan"<br><br>"Tell the woman why and when information about her pregnancy may need to be shared with other agencies."	a. To develop guidelines around data-sharing which are sensitive and proportionate to risk (i.e., between perinatal mental health services and children's social care)<br><br>b. To encourage ongoing communication between agencies after referrals, with feedback and shared decision-making across children's social care and perinatal services.
5. Underfunded services inhibiting good practice	"Healthcare professionals should be given training to ensure they are knowledgeable about safeguarding responsibilities for both the young woman and her unborn baby."	a. Resource perinatal healthcare services with safe staffing levels, sustainable workloads, and sufficient equipment to promote equitable care delivery<br><br>b. Increase training, further education, and supervision capacity for healthcare professionals and encourage career development.

For peer review only

426 ## Strengths and limitations of this study

427 We collected survey data across 8 regions of England and a range of professional
428 groups. Free-text responses allowed professionals to share, in confidence, detailed
429 responses and case examples of service provision and constraints. However, these
430 data are also limited in scope and diversity, given that most respondents were White,

For peer review only - http://bmjopen.bmj.com/site/about/guidelines.xhtml

worked as midwives and were based in Greater London. More detailed understanding of local authority variation in service need and delivery is needed for targeted service improvement.

Conclusion

Women who are at risk of children's social care involvement will likely experience perinatal healthcare inequities. Our findings suggest that current perinatal healthcare provision for this population is inadequate and national guidelines should be updated.

Ethics approval and consent to participate

This study received ethical approval from University College London's Research Ethics Committee (ref: 21605/001).

Competing interests

There are no known conflicts of interests.

Funding

This work was supported by the Economic and Social Research Council UBEL Doctoral Training Programme (ES/P000592/1) and a UCL Culture Engagement Grant. CP, JW, and RL are (in part) supported by the National Institute for Health and Care Research (NIHR) Children and Families Policy Research Unit (PR-PRU-1217-21301). The views expressed are those of the author(s) and not necessarily those of the NIHR or the Department of Health and Social Care.

For peer review only

Author contributions

CG, JW, CP, RB & RL conceived the idea for the study. CG conducted the data collection and analysis. TBM and BLT assisted with data interpretation and manuscript editing. CG drafted the manuscript, and all authors approved the final draft.

Acknowledgements

We would like to thank the healthcare professionals who took the time to complete our online survey. We would also like to acknowledge the support of our project advisory group of women who offered support in the development of this study and in interpreting findings.

References

1. NHS England. nhs.uk. 2020 [cited 2023 Aug 14]. Your NHS pregnancy journey. Available from: https://www.nhs.uk/pregnancy/finding-out/your-nhs-pregnancy-journey/

2. Deierlein AL, Antoniak K, Chan M, Sassano C, Stein CR. Pregnancy-related outcomes among women with physical disabilities: A systematic review. Paediatr Perinat Epidemiol. 2021;35(6):758–78.

3. Tarasoff LA, Ravindran S, Malik H, Salaeva D, Brown HK. Maternal disability and risk for pregnancy, delivery, and postpartum complications: a systematic review and meta-analysis. Am J Obstet Gynecol. 2020 Jan;222(1):27.e1-27.e32.

4. Langham J, Gurol-Urganci I, Muller P, Webster K, Tassie E, Heslin M, et al. Obstetric and neonatal outcomes in pregnant women with and without a history of specialist mental health care: a national population-based cohort study using linked routinely collected data in England. Lancet Psychiatry. 2023 Aug;S2215036623002006.

5. NIDA. National Institute on Drug Abuse. 2023 [cited 2023 Oct 19]. Substance Use While Pregnant and Breastfeeding. Available from: https://nida.nih.gov/publications/research-reports/substance-use-in-women/substance-use-while-pregnant-breastfeeding

6. Conners NA, Bradley RH, Mansell LW, Liu JY, Roberts TJ, Burgdorf K, et al. Children of mothers with serious substance abuse problems: an accumulation of risks. Am J Drug Alcohol Abuse. 2004;30(1):85–100.

7. Harron K, Gilbert R, Fagg J, Guttmann A, van der Meulen J. Associations between pre-pregnancy psychosocial risk factors and infant outcomes: a population-based cohort study in England. Lancet Public Health. 2021;

For peer review only - http://bmjopen.bmj.com/site/about/guidelines.xhtml

8. Mamun A, Biswas T, Scott J, Sly PD, McIntyre HD, Thorpe K, et al. Adverse childhood experiences, the risk of pregnancy complications and adverse pregnancy outcomes: a systematic review and meta-analysis. BMJ Open. 2023 Aug 1;13(8):e063826.

9. Cardwell & Wainwright. Making Better Births a reality for women with multiple disadvantages [Internet]. London: Birth Companions, Revolving Door Agency; 2019 [cited 2023 Nov 1]. Available from: https://www.birthcompanions.org.uk/resources/6-making-better-births-a-reality-for-women-with-multiple-disadvantages

10. Grant C, Radley J, Philip G, Lacey R, Blackburn R, Powell C, et al. Parental health in the context of public family care proceedings: A scoping review of evidence and interventions. Child Abuse Negl. 2023 Jun 1;140:106160.

11. MBRRACE-UK. Saving Lives, Improving Mothers' Care: Lessons learned to inform maternity care from the UK and Ireland Confidential Enquiries into Maternal Deaths and Morbidity 2018-20. [Internet]. Oxford: National Perinatal Epidemiology Unit, University of Oxford; 2022 [cited 2023 Oct 19]. Available from: https://www.npeu.ox.ac.uk/mbrrace-uk/reports

12. Park JM, Solomon P, Mandell DS. Involvement in the child welfare system among mothers with serious mental illness. Psychiatr Serv Wash DC. 2006 Apr;57(4):493–7.

13. Sarkola T, Kahila H, Gissler M, Halmesmäki E. Risk factors for out-of-home custody child care among families with alcohol and substance abuse problems. Acta Paediatr Int J Paediatr. 2007;96(11):1571–6.

14. Booth T, Booth W, McConnell D. The prevalence and outcomes of care proceedings involving parents with learning difficulties in the family courts. J Appl Res Intellect Disabil. 2005;

15. Bacon G, Sweeney A, Batchelor R, Grant C, Mantovani N, Peter S, et al. At the Edge of Care: A systematic review and thematic synthesis of parent and practitioner views and experiences of support for parents with mental health needs and children's social services involvement. Health Soc Care Community. 2023 Apr 17;

16. Roberts SCM, Pies C. Complex calculations: how drug use during pregnancy becomes a barrier to prenatal care. Matern Child Health J. 2011 Apr;15(3):333–41.

17. Canfield M, Radcliffe P, Marlow S, Boreham M, Gilchrist G. Maternal substance use and child protection: a rapid evidence assessment of factors associated with loss of child care. Child Abuse Negl. 2017 Aug;70:11–27.

18. Lever Taylor B, Brobbey L, Howard LM, Masters B, Molloy Z, Potts LC, et al. What Are the Sociodemographic and Clinical Characteristics and Needs of Mothers Who Access Acute Postpartum Psychiatric Care and Have Children's Social Care Involvement? Cooke A, editor. Health Soc Care Community. 2023 Feb 11;2023:1–13.

19. Grant C, Powell C, Philip G, Blackburn R, Lacey R, Woodman J. 'On paper, you're normal': narratives of unseen health needs among women who have had children removed from their care. J Public Health. 2023 Jul 31;fdad137.

For peer review only

For peer review only - http://bmjopen.bmj.com/site/about/guidelines.xhtml

20. Birth Companions. The Birth Charter for women with involvement from children's social care. London; 2023.

21. Braun V, Clarke V, Boulton E, Davey L, McEvoy C. The online survey as a *qualitative* research tool. Int J Soc Res Methodol. 2021 Nov 2;24(6):641–54.

22. Qualtrics. Qualtrics [Internet]. Provo, Utah, USA; 2005. Available from: https://www.qualtrics.com

23. Gale NK, Heath G, Cameron E, Rashid S, Redwood S. Using the framework method for the analysis of qualitative data in multi-disciplinary health research. BMC Med Res Methodol. 2013 Sep 18;13(1):117.

24. MacAlister J. The independent review of children's social care. 2022.

25. Broadhurst K, Mason MC, Bedston S, Alrouh B, Morriss L, McQuarrie MT, et al. Vulnerable birth mothers and recurrent care proceedings. Lanc Univ Lanc. 2017;

26. Bicknell-Morel T. Developing supportive midwifery practice in the context of the removal of a baby at birth. 2021.

27. Care Quality Commission (CQC). Quality of care [Internet]. 2023 [cited 2023 Nov 20]. Available from: https://www.cqc.org.uk/publications/major-report/state-care/2022-2023/quality-of-care#maternity

28. NHS England. National Maternity Review: Better Births Improving outcomes of maternity services in England A Five Year Forward View for maternity care. London: NHS England; 2016.

29. Sandall, J., Coxon, K., Mackintosh, N., Rayment-Jones, H., Locock, L. and Page, L.(writing on behalf of the Sheila Kitzinger symposium). Relationships: the pathway to safe, high-quality maternity care Report from the Sheila Kitzinger symposium at Green Templeton College. Green Templeton College, Oxford; 2016.

30. Weyman AK, Glendinning R, Costa J, O'Hara R, Roy D, Nolan P. Should I stay or should I go? NHS staff retention in the post COVID-19 world: Challenges and prospects [Internet]. 2023 Feb [cited 2023 Nov 23]. Available from: https://pure.ulster.ac.uk/en/publications/1c2fb4df-4877-4936-a28b-5889d030b31f

31. NHS. The NHS long term plan [Internet]. 2019. Available from: https://www.longtermplan.nhs.uk/

32. Wangler S, Streffing J, Simon A, Meyer G, Ayerle GM. Measuring job satisfaction of midwives: A scoping review. PLOS ONE. 2022 Oct 13;17(10):e0275327.

33. Broadhurst K, Mason C. Child removal as the gateway to further adversity: Birth mother accounts of the immediate and enduring collateral consequences of child removal. Qual Soc Work. 2020;

For peer review only

For peer review only - http://bmjopen.bmj.com/site/about/guidelines.xhtml

34. Cès A, Falissard B, Glangeaud-Freudenthal NMC, Sutter-Dallay AL, Gressier F. Pregnancy in women with psychotic disorders: Risk factors associated with mother-baby separation. Arch Womens Ment Health. 2018;21(6):699–706.

35. Rayment-Jones H, Silverio SA, Harris J, Harden A, Sandall J. Project 20: Midwives' insight into continuity of care models for women with social risk factors: what works, for whom, in what circumstances, and how. Midwifery. 2020 May;84:102654.

36. Maternal Mental Health Alliance. Specialist Mental Health Midwives: What they do and why they matter [Internet]. 2013 [cited 2023 Oct 23]. Available from: https://www.rcm.org.uk/publications/publications/specialist-mental-health-midwives-what-they-do-and-why-they-matter/

37. Mason C, Ward H, Broadhurst K. Giving HOPE and minimising trauma: An intervention to support women who are separated from their babies at birth due to safeguarding concerns. Child Abuse Rev. 2023;32(1):e2809.

38. Lushey CJ, Barlow J, Rayns G, Ward H. Assessing Parental Capacity when there are Concerns about an Unborn Child: Pre-Birth Assessment Guidance and Practice in England. Child Abuse Rev. 2018;27(2):97–107.

39. National Collaborating Centre for Women's and Children's Health (UK). Pregnancy and Complex Social Factors: A Model for Service Provision for Pregnant Women with Complex Social Factors [Internet]. London: RCOG Press; 2010 [cited 2023 Aug 14]. (National Institute for Health and Clinical Excellence: Guidance). Available from: http://www.ncbi.nlm.nih.gov/books/NBK62615/

40. Mason C, Broadhurst K, Ward H, Barnett A. Born into Care: Best practice guidelines for when the state intervenes at birth. 2023.

For peer review only

For peer review only - http://bmjopen.bmj.com/site/about/guidelines.xhtml

Supplementary Material 1

Supporting women with complex health needs who might be at risk of children's social care involvement – survey questions

About you
The following questions will give us a better understanding of who you are and your role in maternity services. Please skip any questions you don't feel comfortable answering.

Gender identity
- Male
- Female
- Non-binary
- Other
- Prefer not to say

Age
- 18-14
- 25-29
- 30-24
- 35-39
- 40-44
- 45-49
- 50-54
- 55+
- Prefer not to say

Ethnicity
- Asian/Asian British (Indian, Pakistani, Bangladeshi, Chinese, other Asian background)
- Black, Black British, Caribbean or African (Caribbean, African, Any other Black, Black British, or Caribbean background)
- Mixed or multiple ethnic groups (White and Black Caribbean, White and Black African, White and Asian, Any other Mixed or multiple ethnic background).
- White (English, Welsh, Scottish, Northern Irish or British, Irish, Gypsy or Irish Traveller, Roma, Any other White background)
- Other ethnic group (Arab, Any other ethnic group)
- Prefer not to say

What is your job role within maternity services?
- Midwife
- Health visitor

For peer review only

- Support worker
- Staff nurse
- Neonatal nurse
- Obstetrician
- Perinatal psychologist
- Perinatal psychiatrist
- Other (please specify)

What setting do you work in? (select as many as apply)

- NHS/public sector
- Private sector healthcare
- Third sector/charity organisations

In which region of England do you currently work?

- North West
- North East
- Yorkshire and the Humber
- East Midlands
- West Midlands
- South East
- East of England
- South West
- Greater London

How long have you been in this field?

- Less than 1 year
- 1-2 years
- 2-5 years
- 5-10 years
- 10-15 years
- 15 years+

Briefly describe your job role

E.g. 'I work as a health visitor and support families with a new baby from pregnancy until the child is 5 years old. I've been in the same clinic in Manchester for 10 years and specialise in supporting women breastfeed. I have recently started facilitating a breastfeeding 'drop-in' clinic once a week for mothers.'

For peer review only

Your experiences and attitudes

Chronic physical conditions

We define chronic physical conditions as conditions that last 1 year or more and require ongoing medical attention or limit activities of daily living or both (e.g., epilepsy, asthma, heart-disease).

Have you had experience supporting women with chronic physical conditions in maternity services?

- Yes
- No
- Unsure

If yes, what did you do in your role to support these needs?

What are some of the barriers to providing adequate maternity care to women with chronic physical health conditions?

Tell us how much you agree with the following statements.

I have had appropriate training to know how to support women with chronic physical conditions.

- Strongly disagree
- Disagree
- Neither agree nor disagree
- Agree
- Strongly agree

I feel confident supporting women with chronic physical conditions in maternity services.

- Strongly disagree
- Disagree
- Neither agree nor disagree
- Agree
- Strongly agree

Mental health need

For peer review only

We define mental health need as the presentation, or diagnosis, of symptoms associated with depression, anxiety, panic, mania, delusions, hallucinations or disordered behaviours (i.e., eating, checking, compulsions).

Have you had experience supporting women with mental health needs in maternity services?

- Yes
- No
- Unsure

If yes, what did you do in your role to support their needs?

What are some of the barriers to providing adequate maternity care to women mental health needs?

Tell us how much you agree with the following statements.
I have had appropriate training to know how to support women with mental health needs in maternity services.

- Strongly disagree
- Disagree
- Neither agree nor disagree
- Agree
- Strongly agree

I feel confident supporting women with mental health needs in maternity services.

- Strongly disagree
- Disagree
- Neither agree nor disagree
- Agree
- Strongly agree

Developmental disabilities
We define developmental disabilities as an impairment in one of four areas: physical, learning, language or behaviour. Developmental disabilities are inclusive of intellectual (or learning) disabilities, attention deficit and hyperactivity disorder (ADHD) and autism spectrum disorders (ASD).

For peer review only

Have you had experience supporting women with developmental disabilities in maternity services?

- Yes
- No
- Unsure

If yes, what did you do in your role to support these needs?

What are some of the barriers to providing adequate maternity care to women with developmental disabilities?

Tell us how much you agree with the following statements.

I have had appropriate training to know how to support women with developmental disabilities in maternity services.

- Strongly disagree
- Disagree
- Neither agree nor disagree
- Agree
- Strongly agree

I feel confident supporting women with developmental disabilities in maternity services.

- Strongly disagree
- Disagree
- Neither agree nor disagree
- Agree
- Strongly agree

Substance misuse

We define substance misuse as the use of alcohol, illegal drugs, or over-the-counter or prescription medications in a way that is harmful to the user or those around them.

Have you had experience supporting women with substance misuse challenges in maternity services?

- Yes
- No
- Unsure

If yes, what did you do in your role to support these needs?

For peer review only

What are some of the barriers to providing adequate maternity care to women with substance misuse challenges?

Tell us how much you agree with the following statements.

I have had appropriate training to know how to support women with substance misuse challenges.

- Strongly disagree
- Disagree
- Neither agree nor disagree
- Agree
- Strongly agree

I feel confident supporting women with substance misuse challenges.

- Strongly disagree
- Disagree
- Neither agree nor disagree
- Agree
- Strongly agree

Child protective services

Supporting women at risk of child protection involvement. Child protective services may become involved with a family when there are concerns around parenting capacity, or it is believed that the child is suffering neglect or abuse. The antenatal period is a crucial time for intervention and support for birth mothers at risk of child protection involvement, including those with complex health needs.

Have you had experience supporting women with child protection involvement in maternity services?

- Yes
- No
- Unsure

If yes, what did you do in your role to support these needs?

For peer review only

What are some of the barriers to providing adequate maternity care to women with child protection involvement?

Tell us how much you agree with the following statements.

I have had appropriate training to know how to support women with child protection involvement.

- Strongly disagree
- Disagree
- Neither agree nor disagree
- Agree
- Strongly agree

I feel confident supporting women with substance misuse challenges.

- Strongly disagree
- Disagree
- Neither agree nor disagree
- Agree
- Strongly agree

Example situation

I would like you to think of a time where you felt you might need to make a referral to children's social care [or to your internal child safeguarding team] as a women's pregnancy progressed.

Describe what made you worried about the child and/or family. Please refrain from using patients' names or identifiable features.

Are there any other comments you would like to make about supporting women with complex health needs in maternity services?

For peer review only

End of the survey.

For peer review only

Standards for Reporting Qualitative Research (SRQR)*

http://www.equator-network.org/reporting-guidelines/srqr/

Title and abstract

	Page/line no(s).
**Title** - Concise description of the nature and topic of the study Identifying the study as qualitative or indicating the approach (e.g., ethnography, grounded theory) or data collection methods (e.g., interview, focus group) is recommended	Lines 1-2
**Abstract** - Summary of key elements of the study using the abstract format of the intended publication; typically includes background, purpose, methods, results, and conclusions	Lines 24-46

Introduction

**Problem formulation** - Description and significance of the problem/phenomenon studied; review of relevant theory and empirical work; problem statement	Lines 72-105
**Purpose or research question** - Purpose of the study and specific objectives or questions	Lines 102-105

Methods

**Qualitative approach and research paradigm** - Qualitative approach (e.g., ethnography, grounded theory, case study, phenomenology, narrative research) and guiding theory if appropriate; identifying the research paradigm (e.g., postpositivist, constructivist/ interpretivist) is also recommended; rationale**	Lines 106-110
**Researcher characteristics and reflexivity** - Researchers' characteristics that may influence the research, including personal attributes, qualifications/experience, relationship with participants, assumptions, and/or presuppositions; potential or actual interaction between researchers' characteristics and the research questions, approach, methods, results, and/or transferability	Lines 106-110
**Context** - Setting/site and salient contextual factors; rationale**	Lines 109-116
**Sampling strategy** - How and why research participants, documents, or events were selected; criteria for deciding when no further sampling was necessary (e.g., sampling saturation); rationale**	Lines 109-116
**Ethical issues pertaining to human subjects** - Documentation of approval by an appropriate ethics review board and participant consent, or explanation for lack thereof; other confidentiality and data security issues	Lines 423-425
**Data collection methods** - Types of data collected; details of data collection procedures including (as appropriate) start and stop dates of data collection and analysis, iterative process, triangulation of sources/methods, and modification of procedures in response to evolving study findings; rationale**	Lines 110-111<br>Lines 118-128

For peer review only - http://bmjopen.bmj.com/site/about/guidelines.xhtml

**Data collection instruments and technologies** - Description of instruments (e.g., interview guides, questionnaires) and devices (e.g., audio recorders) used for data collection; if/how the instrument(s) changed over the course of the study	Lines 117-128
**Units of study** - Number and relevant characteristics of participants, documents, or events included in the study; level of participation (could be reported in results)	Lines 146-156
**Data processing** - Methods for processing data prior to and during analysis, including transcription, data entry, data management and security, verification of data integrity, data coding, and anonymization/de-identification of excerpts	Lines 131-141
**Data analysis** - Process by which inferences, themes, etc., were identified and developed, including the researchers involved in data analysis; usually references a specific paradigm or approach; rationale**	Lines 131-141
**Techniques to enhance trustworthiness** - Techniques to enhance trustworthiness and credibility of data analysis (e.g., member checking, audit trail, triangulation); rationale**	Lines 131-141

Results/findings

**Synthesis and interpretation** - Main findings (e.g., interpretations, inferences, and themes); might include development of a theory or model, or integration with prior research or theory	Lines 156-358
**Links to empirical data** - Evidence (e.g., quotes, field notes, text excerpts, photographs) to substantiate analytic findings	Lines 156-358

Discussion

**Integration with prior work, implications, transferability, and contribution(s) to the field -** Short summary of main findings; explanation of how findings and conclusions connect to, support, elaborate on, or challenge conclusions of earlier scholarship; discussion of scope of application/generalizability; identification of unique contribution(s) to scholarship in a discipline or field	Lines 361-410
**Limitations** - Trustworthiness and limitations of findings	Lines 412-418

Other

**Conflicts of interest** - Potential sources of influence or perceived influence on study conduct and conclusions; how these were managed	Lines 426-427
**Funding** - Sources of funding and other support; role of funders in data collection, interpretation, and reporting	Lines 428-434

*The authors created the SRQR by searching the literature to identify guidelines, reporting standards, and critical appraisal criteria for qualitative research; reviewing the reference lists of retrieved sources; and contacting experts to gain feedback. The SRQR aims to improve the transparency of all aspects of qualitative research by providing clear standards for reporting qualitative research.

For peer review only

**The rationale should briefly discuss the justification for choosing that theory, approach, method, or technique rather than other options available, the assumptions and limitations implicit in those choices, and how those choices influence study conclusions and transferability. As appropriate, the rationale for several items might be discussed together.

Reference:
O'Brien BC, Harris IB, Beckman TJ, Reed DA, Cook DA. **Standards for reporting qualitative research: a synthesis of recommendations.** *Academic Medicine*, Vol. 89, No. 9 / Sept 2014
DOI: 10.1097/ACM.0000000000000388

For peer review only

For peer review only - http://bmjopen.bmj.com/site/about/guidelines.xhtml

BMJ Open

Perinatal healthcare for women at risk of children's social care involvement: a qualitative survey of professionals in England

Journal:	*BMJ Open*
Manuscript ID	bmjopen-2023-082914.R1
Article Type:	Original research
Date Submitted by the Author:	16-Feb-2024
Complete List of Authors:	Grant, Claire; University College London Institute of Epidemiology and Health Care, Department of Epidemiology and Public Health<br>Bicknell-Morel, Tamsin; Homerton Healthcare NHS Foundation Trust<br>Lever Taylor, Billie ; King's College London Florence Nightingale Faculty of Nursing Midwifery & Palliative Care, Division of Methodologies<br>Powell, Claire; University College London, Institute of Child Health<br>Blackburn, Ruth; University College London Great Ormond Street Institute of Child Health, Population, Policy and Practice<br>Lacey, Rebecca; St George's University of London, Population Health Research Institute; University College London, Department of Epidemiology and Public Health<br>Woodman, Jenny; University College London Social Research Institute, Thomas Coram Research Institute
**Primary Subject Heading**:	Reproductive medicine
Secondary Subject Heading:	Health policy, Mental health, Public health
Keywords:	MENTAL HEALTH, Maternal medicine < OBSTETRICS, Substance misuse < PSYCHIATRY, Child protection < PAEDIATRICS

SCHOLARONE™
Manuscripts

I, the Submitting Author has the right to grant and does grant on behalf of all authors of the Work (as defined in the below author licence), an exclusive licence and/or a non-exclusive licence for contributions from authors who are: i) UK Crown employees; ii) where BMJ has agreed a CC-BY licence shall apply, and/or iii) in accordance with the terms applicable for US Federal Government officers or employees acting as part of their official duties; on a worldwide, perpetual, irrevocable, royalty-free basis to BMJ Publishing Group Ltd ("BMJ") its licensees and where the relevant Journal is co-owned by BMJ to the co-owners of the Journal, to publish the Work in this journal and any other BMJ products and to exploit all rights, as set out in our licence.

The Submitting Author accepts and understands that any supply made under these terms is made by BMJ to the Submitting Author unless you are acting as an employee on behalf of your employer or a postgraduate student of an affiliated institution which is paying any applicable article publishing charge ("APC") for Open Access articles. Where the Submitting Author wishes to make the Work available on an Open Access basis (and intends to pay the relevant APC), the terms of reuse of such Open Access shall be governed by a Creative Commons licence – details of these licences and which Creative Commons licence will apply to this Work are set out in our licence referred to above.

Other than as permitted in any relevant BMJ Author's Self Archiving Policies, I confirm this Work has not been accepted for publication elsewhere, is not being considered for publication elsewhere and does not duplicate material already published. I confirm all authors consent to publication of this Work and authorise the granting of this licence.

For peer review only

Perinatal healthcare for women at risk of children's social care involvement: a qualitative survey of professionals in England

Author list: Claire Grant[1], Tamsin Bicknell Morel[2], Billie Lever Taylor[3], Claire Powell[4], Ruth Blackburn[4], Rebecca Lacey[1,5], Jenny Woodman[6]

Department of Epidemiology and Public Health, University College London, UK[1]
Homerton Healthcare NHS Foundation Trust, London, UK[2]
Division of Methodologies, Florence Nightingale Faculty of Nursing, Midwifery & Palliative Care, Kings College London[3]
Population, Policy and Practice Department, UCL Great Ormond Street Institute of Child Health, London, UK[4]
Population Health Research Institute, St George's, University of London, London UK[5]
Thomas Coram Research Unit, Social Research Institute, Institute of Education, University College London, UK[6]

Corresponding author

Ms Claire Grant,
Department of Epidemiology and Public Health
University College London
1-19 Torrington Place, London WC1E 6BT
claire.grant.20@ucl.ac.uk

Word count

3,976

Key words

Perinatal health, child protection, maternity

For peer review only

Abstract

Background

Women with complex health needs are more at risk of having children's social care involvement with their newborns than other mothers. Around the time of pregnancy, there are opportunities for health services to support women with these needs and mitigate the risk of mother-baby separation. Yet little is known about healthcare professionals' experiences of providing this support.

Methods

We administered an online survey to perinatal healthcare professionals across England (n=70 responders), including midwives, obstetricians, perinatal psychologists/psychiatrists, and health visitors. We asked about their experiences of providing care for pregnant women with chronic physical conditions, mental health needs, intellectual/developmental disabilities and substance use disorders, who might be at risk of children's social care involvement. We conducted a framework analysis.

Results

We constructed five themes from participant data. These include, 1) inaccessible healthcare for women with complex needs, 2) the challenges and importance of restoring trust, 3) services focusing on individuals, not families, 4) the necessity and caution around multidisciplinary support, and 5) underfunded services inhibiting good practice.

For peer review only

Conclusions

Women who are at risk of children's social care involvement will likely experience perinatal healthcare inequities. Our findings suggest that current perinatal healthcare provision for this population is inadequate and national guidelines need updated to inform support.

For peer review only

For peer review only - http://bmjopen.bmj.com/site/about/guidelines.xhtml

Strengths and limitations of this study

- This study offers a timely insight into lived experiences of providing perinatal healthcare for women at risk of children's social care involvement.
- We collected survey data across different local authorities in England and across a range of professionals who support pregnant women in healthcare.
- Free-text responses allowed professionals to share in-depth responses and case examples of service provision and service constraints.
- Survey methods can be bias towards those who 'self-select' to participate and have a given motive for doing so (e.g., a strong view that they wish to share).
- Findings can contribute towards the development of national guidelines for supporting women at risk of children's social care involvement.

For peer review only

For peer review only - http://bmjopen.bmj.com/site/about/guidelines.xhtml

Background

From conception until one year after birth (the ‘perinatal’ period), mothers in England have various routine contacts with healthcare services. As well as labour and birth support, most women are offered up to 10 antenatal appointments to check the health and development of their babies, in addition to regular postnatal reviews from health visitors, and specialist input, such as from perinatal mental health services, as needed. (1)

Mothers with complex health needs are more likely to have complications during pregnancy and childbirth. (2–4) Newborns are directly affected by parental health and health behaviours, via in utero effects to the developing baby and post-natal caregiving practices. (5) There are also indirect harms of poor parental health on infants, including unstable housing, compromised parental capacity, and increased risk of child abuse/neglect. (6) It is known that histories of trauma, experiences of chronic stress, and psychosocial adversities can impact on women’s experiences of birth and infant outcomes. (7,8) Women who experience severe and multiple disadvantages can be underserved by maternity services and overrepresented in children’s social care. (9,10)

In a 2022 review of perinatal mortality in the UK, 20% of women who died during pregnancy, or in the year following, were found to be involved with children’s social care. (11) Maternal serious mental illness (12), substance use disorder, (13) and intellectual/developmental disability (14), have all been associated with an increased risk of pre-birth assessments and newborn entry into out-of-home care. (10) The fear of children’s social care involvement can itself act as a barrier for parents accessing help from relevant services, including specialist support for their mental health or substance

For peer review only

use. (15,16) Additionally, health needs can co-exist and be compounded by adverse social circumstances, such as low social support and living in poverty. (17,18)

Many of the challenges faced by women with children's social care involvement are the product of lifelong disadvantages and lack of meaningful support (19), factors which long pre-date their pregnancy. (7) There are also various barriers to providing adequate care for this population, including parental distrust of services. (15) Nevertheless, professionals working in perinatal healthcare have opportunities to support women in services, to mitigate some of the health risks associated with children's social care involvement, and to make appropriate referrals for continued support.(20) Our study aims to explore the experiences of perinatal healthcare providers and reflect on how services and systems might better support women at risk of children's social care involvement (i.e., those with 'complex needs').

Methods

Survey methods were chosen due to their ability to efficiently collect qualitative data across settings and populations. The anonymity of completing the survey online was also thought to increase honest accounts of professionals' experiences, attitudes, and beliefs. (21)

Sampling strategy

Healthcare professionals were invited to participate via email, newsletters, and social media posts on Twitter between October-December 2022. The recruitment process utilised existing clinical and academic networks, including maternity interest groups and relevant organisations. Participants had to be employed in a healthcare role within

For peer review only - http://bmjopen.bmj.com/site/about/guidelines.xhtml

For peer review only

perinatal services in England. All responses were voluntary, and consent was sought from each participant. Ethical approval was obtained from UCL Research Ethics Committee (ref: 21605/001) and data were collected via Qualtrics. (22)

Survey questions

We developed our survey questions based on findings from a systematic scoping review into parental health in the context of children's social care. (10) We asked about experiences of supporting women with different types of complex health needs that have been associated with children's social care involvement. These included chronic physical conditions, mental health needs, intellectual/developmental disabilities, and substance use disorders (see Table 1 for definitions). Participants were asked to reflect on their experiences of supporting women with these needs. Most survey questions were free text and had no limit on the length of participants' responses. We asked respondents what they did in their role to support these women and what the barriers to providing care were. We also asked about experiences of safeguarding and referrals to children's social care (see Supplementary Material 1 for survey questions).

Table 1 Definitions of health need

**Mental health need**	The presentation or diagnosis of symptoms associated with depression, anxiety, psychosis, eating disorders, and emotional distress (including post-traumatic stress disorder).
**Developmental disabilities**	An impairment of learning, language, or behaviour, inclusive of intellectual (or learning) disabilities, attention deficit and hyperactivity disorder (ADHD) and autism spectrum disorders (ASD).
**Substance use disorders**	The use of alcohol, illicit drugs or over the counter or prescription medications in a way that is harmful to the user or those around them.

For peer review only - http://bmjopen.bmj.com/site/about/guidelines.xhtml

**Chronic physical conditions**	Conditions that lasted 1 year or more and required ongoing medical attention or limited activities of daily living or both (e.g., epilepsy, permanent injuries).

Analysis

Descriptive statistics of demographic data were calculated using StatsiQ within the Qualtrics software. Free-text responses were treated as qualitative data and analysed using framework analysis. (23) Authors familiarised themselves with the free-text responses and conducted an initial inductive coding exercise with a subset of data. Phrases were labelled to capture meaning or content relevant to the research question. Initial codes were reviewed and used to develop an analytical framework. Once the framework was refined, all free-text responses from the survey were exported into Excel and data organised within the framework to relevant codes. Codes were conceptually organised to construct themes and capture participant experiences.

Patient and public involvement

Researchers presented findings to two women with lived experience of children's social care involvement. We asked women to reflect on our themes and descriptions in relation to their own experiences. Minutes from this meeting was considered part of data interpretation and their commentary is included in the results.

Participants

A total of 70 professionals responded to our online survey, with most participants identifying as female (96%) and White (88%). Half of the respondents worked as midwives (50%) and around half worked in Greater London (46%). See Table 2 for distribution of responders across England. Nearly all had experience of supporting

For peer review only - http://bmjopen.bmj.com/site/about/guidelines.xhtml

women with mental health conditions (96%), and around three quarters had supported mothers with substance use disorders (78%), and intellectual/developmental disabilities (72%). Over half had supported a woman with a chronic physical condition (53%) and nearly all had supported a pregnancy that had children's social care involvement (92%) (See Table 2).

Table 2 Participant demographics

		Count (n)	Percentage (%)
Healthcare professionals			
**Gender**			
	Female	67	96
	Male	3	4
**Age**			
	18-29	19	27
	30-39	18	26
	40-49	17	24
	50+	16	23
**Ethnicity**			
	Asian/Asian British	3	5
	Black, Black British, Caribbean, or African	4	5
	White	61	88
	Prefer not to say	2	2
**Job Role**			
	Midwife	35	50
	Health visitor	8	12
	Nurse or support worker	4	6
	Perinatal psychologist/psychiatrist	7	10
	Other	16	22
**Region of England**			
	North East	1	2
	Yorkshire and the Humber	10	15
	East Midlands	4	6
	West Midlands	11	16
	South East	6	9
	East of England	2	3
	South West	2	3
	Greater London	31	46

For peer review only - http://bmjopen.bmj.com/site/about/guidelines.xhtml

**Years of experience**			
	Less than 1	11	16
	1-5 years	14	20
	5-10 years	20	29
	10-15 years	10	15
	15 years +	10	15
	Prefer not to say	3	5
**Direct experience supporting**			
	Chronic physical conditions	37	53
	Mental health difficulties	67	96
	Substance use disorders	54	78
	Developmental disabilities	50	72
	Child safeguarding concerns	64	92

Results

From participant data, we constructed five themes, 1) inaccessible healthcare for women with complex needs, 2) the challenges and importance of restoring trust, 3) services focusing on individuals, not families, 4) the necessity and caution around multidisciplinary support, and 5) underfunded services inhibiting good practice. Descriptions of each theme and supporting quotations are reported below, alongside commentary from our lived experience group.

1. Inaccessible healthcare for women with complex needs

Participants described challenges providing equitable perinatal healthcare for women with complex needs. They gave examples of women with mental health difficulties, disabilities, and chronic conditions receiving care in universal clinics which were not suitable for their needs.

> "High thresholds for specialist interventions means that the responsibility of ensuring safety of mums and babies is down to us." (ID22, family nurse)

For peer review only

Professionals adjusted care provision as best they could but felt that this was limited without specialist knowledge or facilities. Respondents felt that mothers who faced multiple adversities, including migrant women, often had more complicated pregnancies yet faced additional barriers to care (e.g., cultural, language, and financial).

> “I cared for a woman with a learning disability who was an undocumented migrant…and there was lots of social services involvement around protecting the baby, but none available to support her to keep her child, because of her status.” (ID42, midwife)

Midwives stressed the importance of having interpreters at appointments and using resources to aid communication with mothers who had language or communication impairments.

> “It’s important to use and adapt resources in services, such as birth plans that use images rather than words, or hospital passports to aid communication for those who need it.” (ID47, midwife)

Women’s access to specialist services was inhibited by lack of local resource, high thresholds, and narrow windows of eligibility. For professionals who had experience practicing in different areas, the disparity in care quality across England was noted. Participants also described a rise in the number of women using substances throughout pregnancy. They felt that available support was insufficient, especially given that substance use was a prominent feature of safeguarding and risk reports.

> “There is a real stigma in both the public and also in maternity services for pregnant women who use substances. Some services don’t even have a specialist substance misuse midwife.” (ID07, midwife)

For peer review only - http://bmjopen.bmj.com/site/about/guidelines.xhtml

Respondents reflected on how women who used substances were ineligible for support at a mental health ‘mother and baby unit’ (MBU), despite mental health needs and substance use likely co-existing. Women could also be excluded from perinatal mental health services if they were under 18, had experienced a pregnancy loss (stillbirth, miscarriage, or termination) or had their baby removed at birth.

“My clients are mainly under 18, which means that they are unable to access perinatal mental health services and get placed into CAMHS (child and adolescent mental health services) which is generally inappropriate for their needs.” (ID39, Family Nurse)

Other services, such as health visiting, halted following newborn removal via children’s social care. Professionals commented on this care as fragmented and shortsighted. Our lived experienced group emphasised the importance of being able to access perinatal care, even when those babies were no longer living or had been placed in out of home care.

“The timeframe they have is so short and once discharged have very little parallel planning for future treatment and support.” (ID44, midwife)

Making service provision accessible often required flexibility, such as options for longer or last-minute appointments, communication via SMS, or the offer of home-based support. There was consensus across professional groups that flexible, and women centered perinatal care was best practice, yet professionals reported feasibility challenges implementing such strategies within NHS settings.

2. The challenges and importance of restoring trust

For peer review only - http://bmjopen.bmj.com/site/about/guidelines.xhtml

Respondents stated that building strong relationships with women was key to delivering meaningful care. This included providing emotional support, being an advocate, and treating women with dignity and respect. Our lived experience group felt that professionals should show kindness in their care and be compassionate towards women's circumstances. For psychologists and perinatal mental health therapists, fostering trusted clinical relationships was considered a key feature of treatment and service delivery. Yet this was not always possible for all professionals. Midwives described busy caseloads and staffing issues as barriers to establishing trust with mothers who had complex needs, particularly when there was lack of continuity of care. The impact of a positive relationship on engagement with services and mother/baby outcomes was noted.

"Relationship building is the key. A woman will return for advice if she feels understood and listened to. I am in a much better position to support and advice if a woman trusts me and understands what I say to her." (ID11, health visitor)

For women at risk of children's social care involvement, professionals felt there was an ingrained distrust of services. Respondents reflected on having to restore women's confidence in public services' ability to support them. Considerations of how to build back trust included validating women's experiences, providing a non-judgmental space to listen, and understanding aspects of appointments which might be difficult (e.g., discussing safeguarding concerns and potential outcomes).

"Remaining open and non-judgemental to women. To remain a person that they can trust and to help anchor them into health care services. Support them with social care

For peer review only - http://bmjopen.bmj.com/site/about/guidelines.xhtml

processes whilst giving them realistic expectations of what is likely to happen." (ID62, midwife)

Our lived experience group agreed that honest and clear communication helped with any escalation of safeguarding concerns.

Participants stated the importance of being able to provide support for families and not just appearing to be policing them. They stated that if a family do not feel they are being heard or supported, they will be more likely to withhold information about health behaviours that trigger concern, such as substance use. Yet the support professionals provided was constrained by service rigidity and safeguarding policies. Some midwives provided examples of strained services being risk averse and inhibiting meaningful risk management with families experiencing difficulties. Referrals to internal safeguarding teams, domestic abuse pathways, or children's social care, at times lead to further distrust of services for families already under stress.

"A woman disclosed domestic violence and although I explained that I had to make a referral I was worried that I would lose her trust. This is what happened - she was contacted by social services in a very unhelpful way and was furious. She declined any further care from me." (ID33, midwife)

Responding to safeguarding concerns sensitively and proportionately relied on trusted clinical relationships between professionals, as well as with families.

"Often, we are not getting feedback on safeguarding referrals, the information all seems to flow one way. So, we are not always feeling that our voices, as midwives, are being heard when social services get involved." (ID39, midwife)

For peer review only

3. Services focusing on individuals, not families

Risk to the unborn baby was a main consideration for determining maternal care plans and pathways, often limiting more holistic, family-focused work.

> "Services may or may not take a rounded view of the mother and her child's need." (ID22, psychologist), as "the unborn baby or child's safety can often take priority over efforts to safeguard the family as a whole." (ID54, midwife)

A participant (psychologist) stated that services should support both mother and baby, offering services that target women's needs as a means of enabling caregiving capacity, and therefore supporting newborn safety. Midwives and health visitors commented on the importance of looking to existing networks of support in a woman's life to support risk management. Involving partners was described as integral to crisis planning, yet support became more complicated when needs were interrelated (e.g., domestic abuse or co-habiting drug use). Participants described how household risks were often outside the remit of perinatal services , despite raising safeguarding concerns for the baby.

> "Often safeguarding concerns are related to fathers' needs. A woman's partner having severe mental health problems, not engaging with services, being abusive in the house and the woman being subject to coercive control." (ID62, clinical nurse specialist)

Our lived experience group reflected on how services should be mindful of any potential abuse in households and work to support the whole family in a way that doesn't further the risk to the mother. One of the health visitors commented on the dearth of services available for birth fathers. Whilst, in principle, some perinatal services supported both

For peer review only

parents, all were targeted at mothers and most had little capacity to support the wider family unit.

For some women, their own childhood experiences and relationships with their parents played a negative role in their transition to motherhood. Two of the psychology respondents described the perinatal period as a sensitive time for managing complex trauma and felt services should be equipped to manage this.

"There are not enough resources to help women with severe trauma through the several years needed to make it safe/ viable for them to be good and creative mothers to their children when they had insufficient support themselves when a child." (ID22, psychologist)

These participants recommended that any 'whole family' support considers both families of origin and families of creation. Our lived experience group also commented on services acknowledging children in families who are no longer alive or have been placed in out-of-home care.

4. The necessity and caution around multidisciplinary support

Participants reported liaising and working alongside other professionals to support women with complex needs yet described challenges to shared care plans.

"Social services use a different system to us, so information sharing is not always done timely. It's even difficult to find schedules for all to be available at the same time." (ID03, occupational therapist)

For peer review only - http://bmjopen.bmj.com/site/about/guidelines.xhtml

However, participants cautioned that routine data sharing across sectors could inhibit mothers' engagement with services.

"Pressure from other services, such as children's social care, make it difficult for women to engage meaningfully with mental health services, making the support superficial. There is fear that services feedback to social care and influence child protection proceedings." (ID52, staff nurse)

Participants identified high thresholds for specialist services as a barrier to multidisciplinary working, with referrals being rejected or mothers being added to long waiting lists. Midwives referenced specialist mental health services as especially hard to access, with many women left without support .

"I have had a lot of women struggle to access mental health services due to long waiting lists, sometimes longer than their pregnancies. I have also had many women rejected because their mental health condition was not severe enough." (ID48, midwife)

Liaising with housing, benefits, and similar services was viewed as integral to supporting women's health. Respondents mentioned that local and third sector offerings should be a part of signposting and referral processes. This was corroborated by the lived experience group, who appreciated professionals having local knowledge of organisations that might help specific needs. Yet survey responses illustrated how staff often felt unprepared when supporting women in situations, or with conditions, that they had not received training on. Participants acknowledged that services being overstretched meant less time to dedicate to 'over and above' care, such as building a knowledge base of local provision, learning about more unusual conditions, or engaging

For peer review only

with optional training. This left staff feeling less able to fulfil their role, for example, some health visitors and midwives described being unable to provide breastfeeding advise to women on certain medications.

> “Not everyone understands the full extent of conditions. Staff at services dealing with chronic physical health conditions won't know the impact it has on pregnancy, and maternity staff don't know the full extent of how pregnancy can impact on chronic physical health conditions.” (ID36, occupational therapist)

Our lived experience group spoke about the importance of understanding the ‘whole picture’ and not to simply focus on needs which fit within the remit of a specific service.

5. Underfunded services inhibiting good practice

Most notably, midwives reported insufficient staff levels and an overwhelming number of referrals and appointments. All professionals reported concern around the lack of resourcing for staff and services, alongside an increasing demand of complex pregnancies.

> “Midwives are all incredibly busy due the national shortage of midwives. Women working with children’s social care require additional midwifery input which many feel they do not have capacity to facilitate.” (ID22, midwife)

Midwives also felt that training on specific health needs was insufficient, especially for temporary bank staff brought in due to staffing shortages. One participant reflected on training courses focusing on how to refer to specialist midwives, but not how to support these women in regular antenatal clinics. They reported feeling worried about not knowing how to answer mothers’ questions or how to best tailor care.

For peer review only - http://bmjopen.bmj.com/site/about/guidelines.xhtml

"Having too many babies and women on our caseloads with specialist needs, without being trained to be able to support them. This is a growing area of occurrence and NHS services need to increase their resources and staffing to meet these growing demands." (ID66, midwife)

Staff across professions and services reported high rates of burn out, with unsustainable staffing levels and expectations. Many reflected on how being unable to adequately support mothers had left them feeling frustrated and disheartened. For some respondents, there were comments on how vicarious trauma had impacted on their own wellbeing and ability to fulfil their duties of care.

"It is a very emotionally draining job and often can be very sad. There is a lack of adequate resourcing and capacity issues within the services themselves." (ID08, midwife)

Most participants referenced good leadership, management, and clinical supervision as key components to fostering a healthy workforce.

"Effective supervision is one of the key ways in which we can help to keep babies safe and support their mothers." (ID52, psychologist)

Responses spoke to wider fatigue experienced by public service staff navigating public spending cuts and austerity measures.

For peer review only

Discussion

Our findings describe experiences of perinatal healthcare professionals who support women with complex needs at risk of children's social care involvement. In keeping with wider literature, our results illustrate shortcomings in care provision for women experiencing multiple disadvantages. (9) Given the recent independent review into children's social care, (24) and the push for targeted, early help for vulnerable families, this study offers a timely insight into the role of perinatal services.

Healthcare professionals shared ideas for adaptations and service-level changes that would improve perinatal equity for these mothers, including resourcing professionals to deliver family focused, multidisciplinary care which is compassionate and proportionate to need. This is consistent with what mothers involved with children's social care have reported elsewhere; a need for comprehensive support which addresses multiple disadvantages and enables safe parenting. (20,25,26) Yet despite agreement between mothers and professionals of what 'good' perinatal healthcare looks like, our findings highlight various barriers to implementing this in practice.

Our results indicate that despite the will of individual practitioners, systemic issues within the NHS can result in women at risk of children's social care receiving inadequate perinatal healthcare. (19) Maternity services in England continue to be an area of concern for the Care Quality Commission (CQC), with services and staff under huge pressure to maintain safe clinical standards. (27) In order to provide meaningful support to vulnerable families in the antenatal period, professionals and services must be properly resourced. (28) For example, developing trust with mothers was important for delivering effective care in this context, (29) however professionals described

For peer review only - http://bmjopen.bmj.com/site/about/guidelines.xhtml

challenges doing so in services that were overstretched, understaffed, and managing increasingly complex caseloads. (24) Our results draw attention to the dedication of perinatal healthcare staff who were often doing the best they could, with the resources they had, to meet the needs of vulnerable families. Yet we know that increasing numbers of front-line staff are leaving due to exhaustion and burnout. (30) In keeping with the NHS Long Term Plan, our findings highlight the need to drive recruitment into professional training pathways, such as midwifery and psychology, (31) as well as retain staff in public healthcare, through adequate resourcing, good leadership, and opportunities for professional development. (32)

We found that rigid service structures can leave women who experience multiple disadvantages in the perinatal period falling between the gaps of provision. (9,19) Without targeted intervention during pregnancy, women who are vulnerable to children's social care involvement are not given a fair opportunity to promptly address their difficulties. (9) As illustrated in our findings, this can be frustrating for professionals who often feel unable to provide effective care, or hold family-level risk, within perinatal services. As a result, women can experience fragmented care which is not always responsive to level of need. For example, women being 'left behind' by perinatal services following mother-baby separation, despite the known consequences on maternal health. (10,33,34)

There are local examples of good practice, including continuity of care models, (35) pre-birth support led by specialist midwives, (36) and interventions to support women in perinatal services following newborn separation. (37) However, such initiatives are not underpinned by national policy or guidelines, leading to inconsistent provision for

For peer review only - http://bmjopen.bmj.com/site/about/guidelines.xhtml

women across the country. (38) Previously published antenatal guidelines from 2010 (last reviewed in 2018) offer advice for supporting women with 'complex social factors'. (39) Yet these guidelines do not address multiple and intersectional disadvantages, or the impact of maternal trauma relevant to service provision (9). More recent evidence has indicated that women at risk of children's social care involvement require targeted and specialist perinatal care (20,40). Our results support the need for updated national guidelines. Some recommendations are outlined in Table 3.

Table 3 Suggested updates to NICE guidelines by research theme

Research theme	Current NICE guidelines	Suggested update
1. Inaccessible healthcare for women with complex needs	"At the booking appointment, give the woman a telephone number to enable her to contact a healthcare professional outside of normal working hours, for example the telephone number of the hospital triage contact, the labour ward or the birth centre."	a. Service leads to provide oversight on tailored care provision for women experiencing multiple disadvantages, including flexible delivery and continuity of care<br>b. To deliver specialist perinatal healthcare in circumstances where mothers 'fall between the gaps' of services (e.g., migrant women, mothers <18 years old, those who use substances, or have lost an infant to death or to care).
2. The challenges and importance of restoring trust	"Respect the woman's right to confidentiality and sensitively discuss her fears in a non-judgemental manner."<br>"Addressing women's fears about the involvement of children's services and potential removal of their child, by providing information tailored to their needs"	a. To understand the impact of past and ongoing trauma on families' interactions with services and remain empathetic when addressing non-engagement<br>b. To be transparent about service capabilities and local safeguarding protocols; cultivating systems where vulnerable families feel heard and supported, rather than policed and judged.

For peer review only - http://bmjopen.bmj.com/site/about/guidelines.xhtml

3. Services focusing on individuals, not families	"In order to facilitate discussion of sensitive issues, provide each woman with a one-to-one consultation, without her partner, a family member or a legal guardian present, on at least one occasion."	a. Consider how services and third sector organisations might support 'whole-family' need, including domestic abuse, and birth fathers' mental health or substance use<br><br>b. To work with mothers, and families, to build risk management plans which draw on their strengths and existing networks of support.
4. The necessity and caution around multidisciplinary support	"Consider initiating a multi-agency needs assessment, including safeguarding issues, so that the woman has a coordinated care plan"<br><br>"Tell the woman why and when information about her pregnancy may need to be shared with other agencies."	a. To develop guidelines around data-sharing which are sensitive and proportionate to risk (i.e., between perinatal mental health services and children's social care)<br><br>b. To encourage ongoing communication between agencies after referrals, with feedback and shared decision-making across children's social care and perinatal services.
5. Underfunded services inhibiting good practice	"Healthcare professionals should be given training to ensure they are knowledgeable about safeguarding responsibilities for both the young woman and her unborn baby."	a. Resource perinatal healthcare services with safe staffing levels, sustainable workloads, and sufficient equipment to promote equitable care delivery<br><br>b. Increase training, further education, and supervision capacity for healthcare professionals and encourage career development.

Strengths and limitations of this study

We collected survey data across 8 regions of England and a range of professional groups. Free-text responses allowed professionals to share, in confidence, detailed responses and case examples of service provision and constraints. However, these data are also limited in scope and diversity, given that most respondents were White,

For peer review only - http://bmjopen.bmj.com/site/about/guidelines.xhtml

worked as midwives and were based in Greater London. It should be acknowledged that survey methods can be bias towards those who 'self-select' to participate and have a given motive for doing so (e.g., a strong view that they wish to share). More detailed understanding of experiences across professional groups and local authority areas is needed to inform service improvement in this area.

Conclusion

Women who are at risk of children's social care involvement will likely experience perinatal healthcare inequities. Our findings suggest that current perinatal healthcare provision for this population is inadequate and national guidelines should be updated.

Author contributions

CG, JW, CP, RB & RL conceived the idea for the study. CG conducted the data collection and analysis. TBM and BLT assisted with data interpretation and manuscript editing. CG drafted the manuscript, and all authors approved the final draft.

Ethics approval and consent to participate

This study received ethical approval from University College London's Research Ethics Committee (ref: 21605/001).

Competing interests

There are no known conflicts of interests.

Funding

This work was supported by the Economic and Social Research Council UBEL Doctoral Training Programme (ES/P000592/1) and a UCL Culture Engagement Grant. CP, JW, and RL are (in part) supported by the National Institute for Health and Care Research

For peer review only

(NIHR) Children and Families Policy Research Unit (PR-PRU-1217-21301). The views expressed are those of the author(s) and not necessarily those of the NIHR or the Department of Health and Social Care.

Data sharing statement

No additional data available.

Acknowledgements

We would like to thank the healthcare professionals who took the time to complete our online survey. We would also like to acknowledge the support of our project advisory group of women who offered support in the development of this study and in interpreting findings.

References

1. NHS England. nhs.uk. 2020 [cited 2023 Aug 14]. Your NHS pregnancy journey. Available from: https://www.nhs.uk/pregnancy/finding-out/your-nhs-pregnancy-journey/

2. Deierlein AL, Antoniak K, Chan M, Sassano C, Stein CR. Pregnancy-related outcomes among women with physical disabilities: A systematic review. Paediatr Perinat Epidemiol. 2021;35(6):758–78.

3. Tarasoff LA, Ravindran S, Malik H, Salaeva D, Brown HK. Maternal disability and risk for pregnancy, delivery, and postpartum complications: a systematic review and meta-analysis. Am J Obstet Gynecol. 2020 Jan;222(1):27.e1-27.e32.

4. Langham J, Gurol-Urganci I, Muller P, Webster K, Tassie E, Heslin M, et al. Obstetric and neonatal outcomes in pregnant women with and without a history of specialist mental health care: a national population-based cohort study using linked routinely collected data in England. Lancet Psychiatry. 2023 Aug;S2215036623002006.

5. NIDA. National Institute on Drug Abuse. 2023 [cited 2023 Oct 19]. Substance Use While Pregnant and Breastfeeding. Available from: https://nida.nih.gov/publications/research-reports/substance-use-in-women/substance-use-while-pregnant-breastfeeding

6. Conners NA, Bradley RH, Mansell LW, Liu JY, Roberts TJ, Burgdorf K, et al. Children of mothers with serious substance abuse problems: an accumulation of risks. Am J Drug Alcohol Abuse. 2004;30(1):85–100.

For peer review only

7. Harron K, Gilbert R, Fagg J, Guttmann A, van der Meulen J. Associations between pre-pregnancy psychosocial risk factors and infant outcomes: a population-based cohort study in England. Lancet Public Health. 2021;

8. Mamun A, Biswas T, Scott J, Sly PD, McIntyre HD, Thorpe K, et al. Adverse childhood experiences, the risk of pregnancy complications and adverse pregnancy outcomes: a systematic review and meta-analysis. BMJ Open. 2023 Aug 1;13(8):e063826.

9. Cardwell & Wainwright. Making Better Births a reality for women with multiple disadvantages [Internet]. London: Birth Companions, Revolving Door Agency; 2019 [cited 2023 Nov 1]. Available from: https://www.birthcompanions.org.uk/resources/6-making-better-births-a-reality-for-women-with-multiple-disadvantages

10. Grant C, Radley J, Philip G, Lacey R, Blackburn R, Powell C, et al. Parental health in the context of public family care proceedings: A scoping review of evidence and interventions. Child Abuse Negl. 2023 Jun 1;140:106160.

11. MBRRACE-UK. Saving Lives, Improving Mothers' Care: Lessons learned to inform maternity care from the UK and Ireland Confidential Enquiries into Maternal Deaths and Morbidity 2018-20. [Internet]. Oxford: National Perinatal Epidemiology Unit, University of Oxford; 2022 [cited 2023 Oct 19]. Available from: https://www.npeu.ox.ac.uk/mbrrace-uk/reports

12. Park JM, Solomon P, Mandell DS. Involvement in the child welfare system among mothers with serious mental illness. Psychiatr Serv Wash DC. 2006 Apr;57(4):493–7.

13. Sarkola T, Kahila H, Gissler M, Halmesmäki E. Risk factors for out-of-home custody child care among families with alcohol and substance abuse problems. Acta Paediatr Int J Paediatr. 2007;96(11):1571–6.

14. Booth T, Booth W, McConnell D. The prevalence and outcomes of care proceedings involving parents with learning difficulties in the family courts. J Appl Res Intellect Disabil. 2005;

15. Bacon G, Sweeney A, Batchelor R, Grant C, Mantovani N, Peter S, et al. At the Edge of Care: A systematic review and thematic synthesis of parent and practitioner views and experiences of support for parents with mental health needs and children's social services involvement. Health Soc Care Community. 2023 Apr 17;

16. Roberts SCM, Pies C. Complex calculations: how drug use during pregnancy becomes a barrier to prenatal care. Matern Child Health J. 2011 Apr;15(3):333–41.

17. Canfield M, Radcliffe P, Marlow S, Boreham M, Gilchrist G. Maternal substance use and child protection: a rapid evidence assessment of factors associated with loss of child care. Child Abuse Negl. 2017 Aug;70:11–27.

18. Lever Taylor B, Brobbey L, Howard LM, Masters B, Molloy Z, Potts LC, et al. What Are the Sociodemographic and Clinical Characteristics and Needs of Mothers Who Access Acute Postpartum Psychiatric Care and Have Children's Social Care Involvement? Cooke A, editor. Health Soc Care Community. 2023 Feb 11;2023:1–13.

For peer review only

19. Grant C, Powell C, Philip G, Blackburn R, Lacey R, Woodman J. 'On paper, you're normal': narratives of unseen health needs among women who have had children removed from their care. J Public Health. 2023 Jul 31;fdad137.

20. Birth Companions. The Birth Charter for women with involvement from children's social care. London; 2023.

21. Braun V, Clarke V, Boulton E, Davey L, McEvoy C. The online survey as a *qualitative* research tool. Int J Soc Res Methodol. 2021 Nov 2;24(6):641–54.

22. Qualtrics. Qualtrics [Internet]. Provo, Utah, USA; 2005. Available from: https://www.qualtrics.com

23. Gale NK, Heath G, Cameron E, Rashid S, Redwood S. Using the framework method for the analysis of qualitative data in multi-disciplinary health research. BMC Med Res Methodol. 2013 Sep 18;13(1):117.

24. MacAlister J. The independent review of children's social care. 2022.

25. Broadhurst K, Mason MC, Bedston S, Alrouh B, Morriss L, McQuarrie MT, et al. Vulnerable birth mothers and recurrent care proceedings. Lanc Univ Lanc. 2017;

26. Bicknell-Morel T. Developing supportive midwifery practice in the context of the removal of a baby at birth. 2021.

27. Care Quality Commission (CQC). Quality of care [Internet]. 2023 [cited 2023 Nov 20]. Available from: https://www.cqc.org.uk/publications/major-report/state-care/2022-2023/quality-of-care#maternity

28. NHS England. National Maternity Review: Better Births Improving outcomes of maternity services in England A Five Year Forward View for maternity care. London: NHS England; 2016.

29. Sandall, J., Coxon, K., Mackintosh, N., Rayment-Jones, H., Locock, L. and Page, L.(writing on behalf of the Sheila Kitzinger symposium). Relationships: the pathway to safe, high-quality maternity care Report from the Sheila Kitzinger symposium at Green Templeton College. Green Templeton College, Oxford; 2016.

30. Weyman AK, Glendinning R, Costa J, O'Hara R, Roy D, Nolan P. Should I stay or should I go? NHS staff retention in the post COVID-19 world: Challenges and prospects [Internet]. 2023 Feb [cited 2023 Nov 23]. Available from: https://pure.ulster.ac.uk/en/publications/1c2fb4df-4877-4936-a28b-5889d030b31f

31. NHS. The NHS long term plan [Internet]. 2019. Available from: https://www.longtermplan.nhs.uk/

32. Wangler S, Streffing J, Simon A, Meyer G, Ayerle GM. Measuring job satisfaction of midwives: A scoping review. PLOS ONE. 2022 Oct 13;17(10):e0275327.

For peer review only

33. Broadhurst K, Mason C. Child removal as the gateway to further adversity: Birth mother accounts of the immediate and enduring collateral consequences of child removal. Qual Soc Work. 2020;

34. Cès A, Falissard B, Glangeaud-Freudenthal NMC, Sutter-Dallay AL, Gressier F. Pregnancy in women with psychotic disorders: Risk factors associated with mother-baby separation. Arch Womens Ment Health. 2018;21(6):699–706.

35. Rayment-Jones H, Silverio SA, Harris J, Harden A, Sandall J. Project 20: Midwives' insight into continuity of care models for women with social risk factors: what works, for whom, in what circumstances, and how. Midwifery. 2020 May;84:102654.

36. Maternal Mental Health Alliance. Specialist Mental Health Midwives: What they do and why they matter [Internet]. 2013 [cited 2023 Oct 23]. Available from: https://www.rcm.org.uk/publications/publications/specialist-mental-health-midwives-what-they-do-and-why-they-matter/

37. Mason C, Ward H, Broadhurst K. Giving HOPE and minimising trauma: An intervention to support women who are separated from their babies at birth due to safeguarding concerns. Child Abuse Rev. 2023;32(1):e2809.

38. Lushey CJ, Barlow J, Rayns G, Ward H. Assessing Parental Capacity when there are Concerns about an Unborn Child: Pre-Birth Assessment Guidance and Practice in England. Child Abuse Rev. 2018;27(2):97–107.

39. National Collaborating Centre for Women's and Children's Health (UK). Pregnancy and Complex Social Factors: A Model for Service Provision for Pregnant Women with Complex Social Factors [Internet]. London: RCOG Press; 2010 [cited 2023 Aug 14]. (National Institute for Health and Clinical Excellence: Guidance). Available from: http://www.ncbi.nlm.nih.gov/books/NBK62615/

40. Mason C, Broadhurst K, Ward H, Barnett A. Born into Care: Best practice guidelines for when the state intervenes at birth. 2023.

For peer review only

For peer review only - http://bmjopen.bmj.com/site/about/guidelines.xhtml

Supplementary Material 1

Supporting women with complex health needs who might be at risk of children's social care involvement – survey questions

About you

The following questions will give us a better understanding of who you are and your role in maternity services. Please skip any questions you don't feel comfortable answering.

Gender identity

- Male
- Female
- Non-binary
- Other
- Prefer not to say

Age

- 18-14
- 25-29
- 30-24
- 35-39
- 40-44
- 45-49
- 50-54
- 55+
- Prefer not to say

Ethnicity

- Asian/Asian British (Indian, Pakistani, Bangladeshi, Chinese, other Asian background)
- Black, Black British, Caribbean or African (Caribbean, African, Any other Black, Black British, or Caribbean background)
- Mixed or multiple ethnic groups (White and Black Caribbean, White and Black African, White and Asian, Any other Mixed or multiple ethnic background).
- White (English, Welsh, Scottish, Northern Irish or British, Irish, Gypsy or Irish Traveller, Roma, Any other White background)
- Other ethnic group (Arab, Any other ethnic group)
- Prefer not to say

What is your job role within maternity services?

- Midwife
- Health visitor
- Support worker

For peer review only

- Staff nurse
- Neonatal nurse
- Obstetrician
- Perinatal psychologist
- Perinatal psychiatrist
- Other (please specify)

What setting do you work in? (select as many as apply)

- NHS/public sector
- Private sector healthcare
- Third sector/charity organisations

In which region of England do you currently work?

- North West
- North East
- Yorkshire and the Humber
- East Midlands
- West Midlands
- South East
- East of England
- South West
- Greater London

How long have you been in this field?

- Less than 1 year
- 1-2 years
- 2-5 years
- 5-10 years
- 10-15 years
- 15 years+

Briefly describe your job role

E.g. 'I work as a health visitor and support families with a new baby from pregnancy until the child is 5 years old. I've been in the same clinic in Manchester for 10 years and specialise in supporting women breastfeed. I have recently started facilitating a breastfeeding 'drop-in' clinic once a week for mothers.'

For peer review only

Your experiences and attitudes

Chronic physical conditions
We define chronic physical conditions as conditions that last 1 year or more and require ongoing medical attention or limit activities of daily living or both (e.g., epilepsy, asthma, heart-disease).

Have you had experience supporting women with chronic physical conditions in maternity services?

- Yes
- No
- Unsure

If yes, what did you do in your role to support these needs?

What are some of the barriers to providing adequate maternity care to women with chronic physical health conditions?

Tell us how much you agree with the following statements.

I have had appropriate training to know how to support women with chronic physical conditions.

- Strongly disagree
- Disagree
- Neither agree nor disagree
- Agree
- Strongly agree

I feel confident supporting women with chronic physical conditions in maternity services.

- Strongly disagree
- Disagree
- Neither agree nor disagree

For peer review only

- Agree
- Strongly agree

Mental health need

We define mental health need as the presentation, or diagnosis, of symptoms associated with depression, anxiety, panic, mania, delusions, hallucinations or disordered behaviours (i.e., eating, checking, compulsions).

Have you had experience supporting women with mental health needs in maternity services?

- Yes
- No
- Unsure

If yes, what did you do in your role to support their needs?

What are some of the barriers to providing adequate maternity care to women mental health needs?

Tell us how much you agree with the following statements.

I have had appropriate training to know how to support women with mental health needs in maternity services.

- Strongly disagree
- Disagree
- Neither agree nor disagree
- Agree
- Strongly agree

I feel confident supporting women with mental health needs in maternity services.

- Strongly disagree
- Disagree
- Neither agree nor disagree
- Agree
- Strongly agree

For peer review only

Developmental disabilities

We define developmental disabilities as an impairment in one of four areas: physical, learning, language or behaviour. Developmental disabilities are inclusive of intellectual (or learning) disabilities, attention deficit and hyperactivity disorder (ADHD) and autism spectrum disorders (ASD).

Have you had experience supporting women with developmental disabilities in maternity services?

- Yes
- No
- Unsure

If yes, what did you do in your role to support these needs?

What are some of the barriers to providing adequate maternity care to women with developmental disabilities?

Tell us how much you agree with the following statements.

I have had appropriate training to know how to support women with developmental disabilities in maternity services.

- Strongly disagree
- Disagree
- Neither agree nor disagree
- Agree
- Strongly agree

I feel confident supporting women with developmental disabilities in maternity services.

- Strongly disagree
- Disagree
- Neither agree nor disagree
- Agree
- Strongly agree

Substance misuse

For peer review only

We define substance misuse as the use of alcohol, illegal drugs, or over-the-counter or prescription medications in a way that is harmful to the user or those around them. Have you had experience supporting women with substance misuse challenges in maternity services?

- Yes
- No
- Unsure

If yes, what did you do in your role to support these needs?

What are some of the barriers to providing adequate maternity care to women with substance misuse challenges?

Tell us how much you agree with the following statements.

I have had appropriate training to know how to support women with substance misuse challenges.

- Strongly disagree
- Disagree
- Neither agree nor disagree
- Agree
- Strongly agree

I feel confident supporting women with substance misuse challenges.

- Strongly disagree
- Disagree
- Neither agree nor disagree
- Agree
- Strongly agree

Child protective services

Supporting women at risk of child protection involvement. Child protective services may become involved with a family when there are concerns around parenting capacity, or it is believed that the child is suffering neglect or abuse. The antenatal

For peer review only

period is a crucial time for intervention and support for birth mothers at risk of child protection involvement, including those with complex health needs.

Have you had experience supporting women with child protection involvement in maternity services?

- Yes
- No
- Unsure

If yes, what did you do in your role to support these needs?

What are some of the barriers to providing adequate maternity care to women with child protection involvement?

Tell us how much you agree with the following statements.

I have had appropriate training to know how to support women with child protection involvement.

- Strongly disagree
- Disagree
- Neither agree nor disagree
- Agree
- Strongly agree

I feel confident supporting women with substance misuse challenges.

- Strongly disagree
- Disagree
- Neither agree nor disagree
- Agree
- Strongly agree

Example situation

I would like you to think of a time where you felt you might need to make a referral to children's social care [or to your internal child safeguarding team] as a women's pregnancy progressed.

For peer review only

Describe what made you worried about the child and/or family. Please refrain from using patients' names or identifiable features.

Are there any other comments you would like to make about supporting women with complex health needs in maternity services?

End of the survey.

For peer review only

Standards for Reporting Qualitative Research (SRQR)*

http://www.equator-network.org/reporting-guidelines/srqr/

Page/line no(s).

Title and abstract

**Title** - Concise description of the nature and topic of the study Identifying the study as qualitative or indicating the approach (e.g., ethnography, grounded theory) or data collection methods (e.g., interview, focus group) is recommended	Lines 1-2
**Abstract** - Summary of key elements of the study using the abstract format of the intended publication; typically includes background, purpose, methods, results, and conclusions	Lines 24-46

Introduction

**Problem formulation** - Description and significance of the problem/phenomenon studied; review of relevant theory and empirical work; problem statement	Lines 72-105
**Purpose or research question** - Purpose of the study and specific objectives or questions	Lines 102-105

Methods

**Qualitative approach and research paradigm** - Qualitative approach (e.g., ethnography, grounded theory, case study, phenomenology, narrative research) and guiding theory if appropriate; identifying the research paradigm (e.g., postpositivist, constructivist/ interpretivist) is also recommended; rationale**	Lines 106-110
**Researcher characteristics and reflexivity** - Researchers' characteristics that may influence the research, including personal attributes, qualifications/experience, relationship with participants, assumptions, and/or presuppositions; potential or actual interaction between researchers' characteristics and the research questions, approach, methods, results, and/or transferability	Lines 106-110
**Context** - Setting/site and salient contextual factors; rationale**	Lines 109-116
**Sampling strategy** - How and why research participants, documents, or events were selected; criteria for deciding when no further sampling was necessary (e.g., sampling saturation); rationale**	Lines 109-116
**Ethical issues pertaining to human subjects** - Documentation of approval by an appropriate ethics review board and participant consent, or explanation for lack thereof; other confidentiality and data security issues	Lines 423-425
**Data collection methods** - Types of data collected; details of data collection procedures including (as appropriate) start and stop dates of data collection and analysis, iterative process, triangulation of sources/methods, and modification of procedures in response to evolving study findings; rationale**	Lines 110-111<br>Lines 118-128

For peer review only - http://bmjopen.bmj.com/site/about/guidelines.xhtml

**Data collection instruments and technologies** - Description of instruments (e.g., interview guides, questionnaires) and devices (e.g., audio recorders) used for data collection; if/how the instrument(s) changed over the course of the study	Lines 117-128
**Units of study** - Number and relevant characteristics of participants, documents, or events included in the study; level of participation (could be reported in results)	Lines 146-156
**Data processing** - Methods for processing data prior to and during analysis, including transcription, data entry, data management and security, verification of data integrity, data coding, and anonymization/de-identification of excerpts	Lines 131-141
**Data analysis** - Process by which inferences, themes, etc., were identified and developed, including the researchers involved in data analysis; usually references a specific paradigm or approach; rationale**	Lines 131-141
**Techniques to enhance trustworthiness** - Techniques to enhance trustworthiness and credibility of data analysis (e.g., member checking, audit trail, triangulation); rationale**	Lines 131-141

Results/findings

**Synthesis and interpretation** - Main findings (e.g., interpretations, inferences, and themes); might include development of a theory or model, or integration with prior research or theory	Lines 156-358
**Links to empirical data** - Evidence (e.g., quotes, field notes, text excerpts, photographs) to substantiate analytic findings	Lines 156-358

Discussion

**Integration with prior work, implications, transferability, and contribution(s) to the field -** Short summary of main findings; explanation of how findings and conclusions connect to, support, elaborate on, or challenge conclusions of earlier scholarship; discussion of scope of application/generalizability; identification of unique contribution(s) to scholarship in a discipline or field	Lines 361-410
**Limitations** - Trustworthiness and limitations of findings	Lines 412-418

Other

**Conflicts of interest** - Potential sources of influence or perceived influence on study conduct and conclusions; how these were managed	Lines 426-427
**Funding** - Sources of funding and other support; role of funders in data collection, interpretation, and reporting	Lines 428-434

*The authors created the SRQR by searching the literature to identify guidelines, reporting standards, and critical appraisal criteria for qualitative research; reviewing the reference lists of retrieved sources; and contacting experts to gain feedback. The SRQR aims to improve the transparency of all aspects of qualitative research by providing clear standards for reporting qualitative research.

For peer review only

For peer review only - http://bmjopen.bmj.com/site/about/guidelines.xhtml

**The rationale should briefly discuss the justification for choosing that theory, approach, method, or technique rather than other options available, the assumptions and limitations implicit in those choices, and how those choices influence study conclusions and transferability. As appropriate, the rationale for several items might be discussed together.

Reference:

O'Brien BC, Harris IB, Beckman TJ, Reed DA, Cook DA. **Standards for reporting qualitative research: a synthesis of recommendations.** *Academic Medicine*, Vol. 89, No. 9 / Sept 2014 DOI: 10.1097/ACM.0000000000000388

For peer review only

For peer review only - http://bmjopen.bmj.com/site/about/guidelines.xhtml
